# Optimization of inelastic multistory structures under seismic vibrations using shape-memory-alloy material

**DOI:** 10.1038/s41598-022-20537-5

**Published:** 2022-10-07

**Authors:** Assaf Shmerling, Matthias Gerdts

**Affiliations:** 1 Department of Civil and Environmental Engineering, Ben-Gurion University of the Negev, 84105 Beer-Sheva, Israel; 2 Institute of Applied Mathematics and Scientific Computing, Universität der Bundeswehr München, 85577 Neubiberg, Germany

**Keywords:** Civil engineering, Mechanical engineering, Engineering

## Abstract

This paper develops a novel optimization methodology for designing Shape-memory-alloy resisting devices (SMARDs) and optimally allocating them to inelastic multistory structures. The solution algorithm is a control gains optimization procedure that refers to a formal optimization problem with an objective function subject to the state-space equation and design limitations. The objective function integrates the squared state components in time, and the state-space equation consists of a newly introduced state vector form that reflects the system's inelasticity. The control gains are the number of total Shape-memory-alloy (SMA) wires attached to the devices in each story, and the design limitations dictate the minimum/maximum number of wires. The solution algorithm consists of five iterative steps that employ the defined Hamiltonian gradients in state and gains and cater to the necessary optimality conditions. The numerical example deals with upgrading an eight-story shear-type frame system. It studies the algorithm efficiency and elaborates on the effect of the optimal weighting matrix by investigating three different configurations. In all cases, the algorithm improves the system's inelastic seismic response—showcasing the reliability of the developed design methodology and the utilization of SMA material.

## Introduction

The shape memory alloy (SMA) material under increased tensile loading is characterized by three main phases: (i) the austenite phase with a linear-elastic strain rate, which extends between zero strain and the yield strain, (ii) the transition phase, in which the material undergoes significant strain due to relatively low tensile loading, and (iii) the martensite phase, in which the material is strengthened, which extends from the second yield to either removal of the load, or failure, or temperature change. Once the tensile loading is removed, the axial stress in the material returns through all the phases it underwent until it reaches zero strain—making the SMA material an efficient superelastic means for vibrations mitigation and structural retrofit.

Various base-isolation systems and energy dissipation devices employ the SMA superelasticity to enhance their capabilities. Khodaverdian et al.^1^ present their base isolation system based on sliding bearings equipped with shape memory alloy. Kumbhar et al.^2^ integrate the SMA with magnetorheological elastomer into a tuned vibration absorber system. Yang et al.^3^ illustrate the possibility of using SMA actuators as tendons to mitigate the vibration of a flexible cantilever beam. Ozbulut and Hurlebaus^4^ compare two SMA-based isolation systems for bridges, exemplifying their effectiveness in limiting the deck's maximum drift. Gur et al.^5^ propose installing SMA material in a liquid column damper by replacing the compliant device with an SMA spring. Shinozuka et al.^6^ employed a lead-rubber-bearing base isolation system supplemented with SMA wires to reduce the isolator's lateral displacement.

The SMA material is also applied to the load carrying systems of civil structures retrofitted against seismic action. Janke et al.^7^ review the earlier applications, such as the bell tower of the Church of San Giorgio (Italy) and the Basilica San Francesco in Assisi (Italy), where the SMA material increases the restoring capabilities of both structures. Dong et al.^8^ expand on installing SMA-based devices in existing bridges by classifying the different applications into five categories: prevention of unseating bridge spans, seismic design of bridge bearings, cable-stayed bridges vibration mitigation, supporting the bridge columns, and bridge-beam applications. Li et al.^9^ further examine the concept of SMA applications in bridges in terms of life cycle and favors their utilization. Rele et al.^10^ proposed taking advantage of bridge rocking isolation by having pier footing, which teeters on elastomeric pads and external restrainers provided by SMA bars to reduce the horizontal pier displacements. Cao and Yi^11^ introduce the SMA spring-damper system and suggest installing it in bridges with laminated rubber bearings, which would slide under seismic excitations. Vůjtěch et al.^12^ present the first application of iron-based SMA bars and implement their use to strengthen the 113-years-old historic roadway bridge in Petrov nad Desnou (Czech Republic). Han et al.^13^ have discussed using SMA wires to control the vibrations of multistory frame structures based solely on their damping capabilities in terms of equivalent high-level damping ratio. Mondal et al.^14^ propose using lead rubber bearings supplemented with SMA-based devices to reduce vibrations of frame structures.

The design methodologies of SMA-based devices usually apply optimization techniques to optimize the coefficients (gains) of an employed stress–strain model or its temperature variable. For example, Bubner et al.^15^ present their Control by Elongation solution procedure and use it to yield the optimal absolute temperature. Piccirillo et al.^16^ address the problem scheme of chaos control theory and solve it using the Riccati equation to determine the SMA oscillator model's optimal gains (coefficients). Zuo et al.^17^ correlate the linear quadratic regulator objective function and minimization of virtual work and use Hamilton's principle to minimize the objective function and calculate the optimal SMA control force gains. Ozbulut et al.^18^ employ a non-dominated sorting genetic algorithm with controlled elitism to optimally allocate SMA damping devices and bracing systems to frame structures to reduce their maximum interstory drifts and absolute accelerations. Das and Mishra^19^ address lead-rubber-bearing supplemented with SMA and optimize the SMA's transformation strength to minimize the acceleration covariance using the MATLAB© toolbox. The authors propose using a genetic algorithm when the MATLAB© toolbox is unavailable. Hassanzadeh and Moradi^20^ used discrete topology optimization in finding the optimal placement and length of SMA braces and the optimal cross-sections of structural members.

Mulay and Shmerling^21^ deal with allocating SMA resisting devices (SMARDs) consisting of inner and outer steel tubes connected by SMA wire loops to regulate the seismic vibration of three-dimensional frame structures. They propose a transfer function matrix that correlates to the inelasticity of a frame structure assigned with SMARDs and minimize the gains of that matrix using a search technique. Das and Tesfamariam^22^ introduce SMA material to the outrigger structure connecting a core structure and perimeter column and seek the optimal location in height to minimize the displacement covariance. Das and Tesfamariam also employed multiobjective optimization in^23^ to address tall-timber buildings with an outrigger system. Mirzai et al.^24^ attempt to mitigate the combination of peak inter-story drift, residual drift, and floor acceleration by adjusting the SMA cross-sectional area, friction force, and stiffness using the cuckoo search algorithm. Chang et al.^25^ introduce their self-centering friction damper and use a genetic algorithm to determine the maximum energy dissipation and equivalent viscous damping. Mirzai et al.^24^ propose the recentering shear damper, whose assembly also consists of an SMA plate, and suggest finding the optimal damper parameters by addressing a lower bound and upper bound optimization problem.

This paper presents a new approach for optimally allocating SMARDs to inelastic frame structures and optimizing their design parameters (number of SMA wires). The methodology differs from existing methods by not relying on genetic/search algorithms^18,19,22,23^, linearization^16,17,20^, or stochastic analysis^19,21^. Instead, the design procedure relies on deterministic analysis and computes the structure's inelastic earthquake response to determine the objective function's steepest gradient. The optimization strategy is suitable for other types of design parameters such as wires area, initial wire length, etc.

## Inelastic system

### Dynamic stability

**Figure 1:**
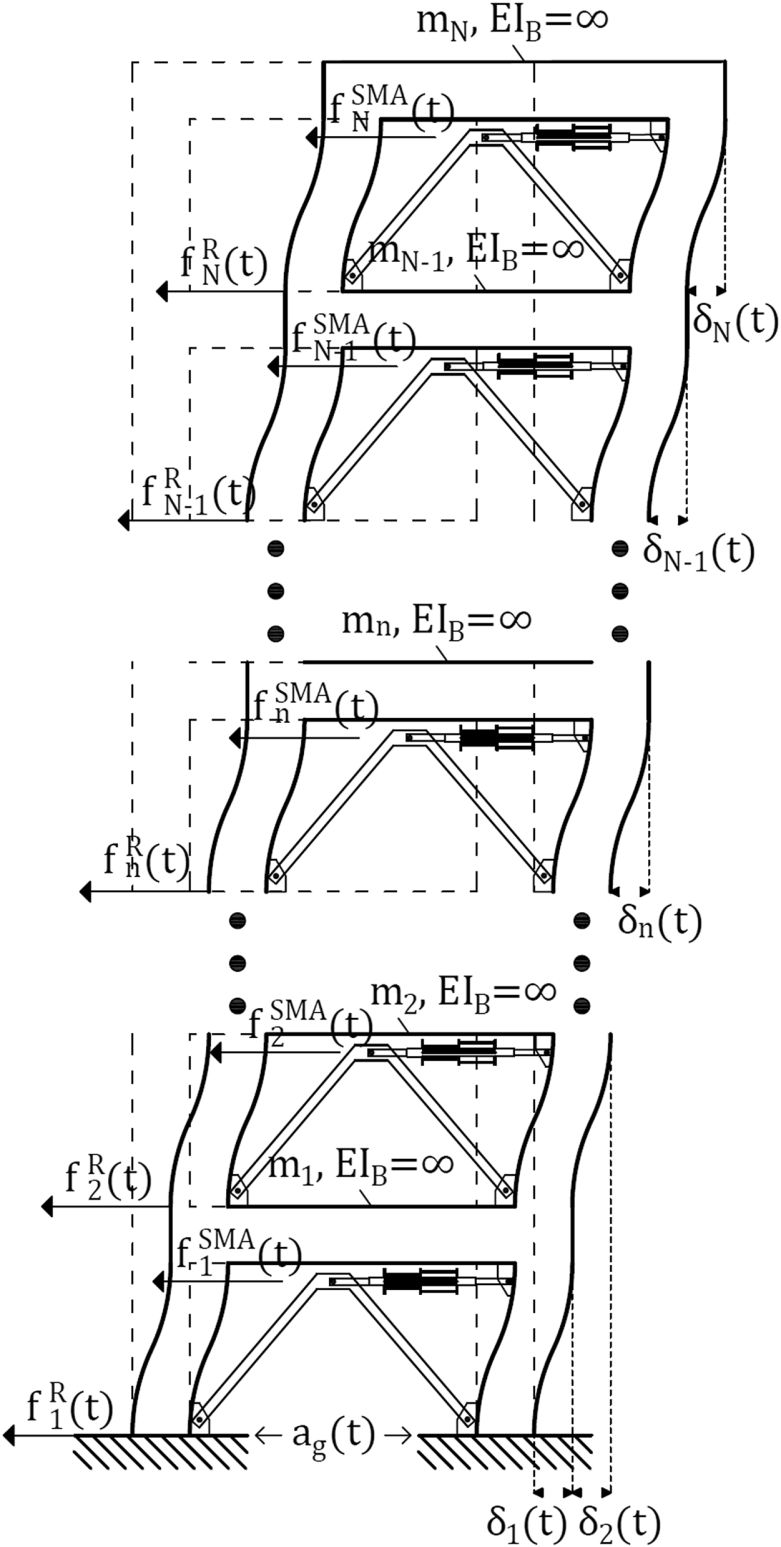
Elevation scheme of a shear-type frame system that undergoes lateral deformations.

The equation representing the dynamic equilibrium of the shear-type frame system is the well-known equation of motion added by the inelastic resisting force introduced by the columns and the nonlinear SMARD force. Figure 1 depicts the elevation scheme of the frame system subject to lateral interstory drift deformations, denoted by ${\delta }_{n}$, and illustrates the consequent resisting shear force and the SMARD force denoted by ${f}_{n}^{R}$ and ${f}_{n}^{SMA}$, respectively. The equation-of-motion related to the addressed system is given by:

1 $$\begin{array}{l}\mathbf{m} \ddot{{\varvec{\updelta}}}\left(\mathrm{t}\right)+\mathbf{c} \dot{{\varvec{\updelta}}}\left(\mathrm{t}\right)+{\mathbf{f}}^{\mathrm{R}}\left({\dot{\mathbf{f}}}^{\mathrm{H}},\mathrm{t}\right)+{\mathbf{f}}^{\mathrm{SMA}}\left({\varvec{\updelta}},\dot{{\varvec{\updelta}}},\mathrm{t}\right)=-\mathbf{m}{\mathbf{e}}_{1}{\mathrm{a}}_{\mathrm{g}}\left(\mathrm{t}\right)\\ \mathrm{and}:\\ {\mathbf{f}}^{\mathrm{R}}\left({\dot{\mathbf{f}}}^{\mathrm{H}},\mathrm{t}\right)=\mathbf{a} {\mathbf{k}}^{\mathrm{el}}{\varvec{\updelta}}\left(\mathrm{t}\right)+{\mathbf{f}}^{\mathrm{H}}\left({\dot{\mathbf{f}}}^{\mathrm{H}},\mathrm{t}\right)\end{array}$$

where ${\mathbf{f}}^{\mathrm{R}}\left({\dot{\mathbf{f}}}^{\mathrm{H}},\mathrm{t}\right)$ is the *N*-dimensional resisting shear force vector, ${\mathbf{f}}^{\mathrm{SMA}}\left({\varvec{\updelta}},\dot{{\varvec{\updelta}}},\mathrm{t}\right)$ is the *N*-dimensional SMARD force vector, ${\varvec{\updelta}}\left(\mathrm{t}\right)$ is the *N*-dimensional interstory drifts vector, $\dot{{\varvec{\updelta}}}\left(\mathrm{t}\right)$ is the *N*-dimensional drift velocities vector, $\ddot{{\varvec{\updelta}}}\left(\mathrm{t}\right)$ is the *N*-dimensional drift accelerations vector, $\mathbf{c}$ is the *N* × *N* inherent Caughey damping matrix, $\mathbf{a}$ is a *N* × *N* diagonal matrix composed of the ratios of post-yield to pre-yield stiffness, ${\mathbf{e}}_{1}$ is an *N*-dimensional vector whose all components are zero except for the first component, which is 1.0, ${a}_{g}\left(t\right)$ is the ground acceleration, $\mathbf{m}$ is the *N* × *N* mass matrix related to the interstory drift accelerations:

2 $$\mathbf{m}=\left[\begin{array}{ccc}1& \dots & 1\\ & \ddots & \vdots \\ 0& & 1\end{array}\right]\left[\begin{array}{ccc}{\mathrm{m}}_{1}& & \\ & \ddots & \\ & & {\mathrm{m}}_{\mathrm{N}}\end{array}\right]\left[\begin{array}{ccc}1& & 0\\ \vdots & \ddots & \\ 1& \cdots & 1\end{array}\right]$$

And ${\mathbf{k}}^{\mathrm{el}}$ is the *N* × *N* elastic-range stiffness matrix composed of the stories' initial stiffness quantity:

3 $${\mathbf{k}}^{\mathrm{el}}=\left[\begin{array}{ccc}{\mathrm{k}}_{1}^{\mathrm{el}}& & \\ & \ddots & \\ & & {\mathrm{k}}_{\mathrm{N}}^{\mathrm{el}}\end{array}\right]$$

The *N*-dimensional force vectors ${\mathbf{f}}^{\mathrm{H}}\left({\dot{\mathbf{f}}}^{\mathrm{H}},\mathrm{t}\right)$ and $\mathbf{a} {\mathbf{k}}^{\mathrm{el}}{\varvec{\updelta}}\left(\mathrm{t}\right)$ are the hysteretic portion and elastic portion of ${\mathbf{f}}^{\mathrm{R}}\left({\dot{\mathbf{f}}}^{\mathrm{H}},\mathrm{t}\right)$ respectively, and ${\mathbf{f}}^{\mathrm{SMA}}\left({\varvec{\updelta}},\dot{{\varvec{\updelta}}},\mathrm{t}\right)$ is *N*-dimensional SMARD shear forces vector. The combination of ${\mathbf{f}}^{\mathrm{R}}\left({\dot{\mathbf{f}}}^{\mathrm{H}},\mathrm{t}\right)$ and ${\mathbf{f}}^{\mathrm{SMA}}\left({\varvec{\updelta}},\dot{{\varvec{\updelta}}},\mathrm{t}\right)$ yields the total shear forces transmitted to the story's columns:

4 $$\mathbf{V}\left(\mathrm{t}\right)={\mathbf{f}}^{\mathrm{R}}\left({\dot{\mathbf{f}}}^{\mathrm{H}},\mathrm{t}\right)+{\mathbf{f}}^{\mathrm{SMA}}\left({\varvec{\updelta}},\dot{{\varvec{\updelta}}},\mathrm{t}\right)$$

The smooth hysteretic model for deteriorating inelastic structures by Wang et al.^26^ is employed in addressing the cyclic behavior of ${\mathbf{f}}^{\mathrm{H}}\left({\dot{\mathbf{f}}}^{\mathrm{H}},\mathrm{t}\right)$, and the modified constitutive law for SMA material by Wilde et al.^27^ is used in modeling ${\mathbf{f}}^{\mathrm{SMA}}\left({\varvec{\updelta}},\dot{{\varvec{\updelta}}},\mathrm{t}\right)$. The SMA hysteretic model is elaborated in the sub-section that follows.

The smooth hysteretic model of Wang et al.^26^ stems from the Bouc-Wen model. Accordingly, the force law refers to the hysteretic portion's Yank (the rate of change of force), that is:

5 $${\dot{\mathbf{f}}}^{\mathrm{H}}\left({\mathbf{f}}^{\mathrm{H}},\dot{{\varvec{\updelta}}}\right)={\mathbf{k}}^{\mathrm{H}}\left({\mathbf{f}}^{\mathrm{H}},\dot{{\varvec{\updelta}}}\right) \dot{{\varvec{\updelta}}}\left(\mathrm{t}\right)$$

where ${\mathbf{k}}^{\mathrm{H}}\left({\mathbf{f}}^{\mathrm{H}},\dot{{\varvec{\updelta}}}\right)$ is the *N* × *N* diagonal tangent stiffness matrix related to ${\mathbf{f}}^{\mathrm{H}}\left({\dot{\mathbf{f}}}^{\mathrm{H}},\mathrm{t}\right)$ and is composed of the stories' lateral hysteretic stiffness portions:

6 $${\mathbf{k}}^{\mathrm{H}}\left({\mathbf{f}}^{\mathrm{H}},\dot{{\varvec{\updelta}}}\right)=\left[\begin{array}{ccc}{\mathrm{k}}_{1}^{\mathrm{H}}\left({\mathrm{f}}_{1}^{\mathrm{H}}, {\dot{\updelta }}_{1}\right)& & \\ & \ddots & \\ & & {\mathrm{k}}_{\mathrm{N}}^{\mathrm{H}}\left({\mathrm{f}}_{\mathrm{N}}^{\mathrm{H}}, {\dot{\updelta }}_{\mathrm{N}}\right)\end{array}\right]$$

Accordingly, ${\mathbf{f}}^{\mathrm{H}}\left({\dot{\mathbf{f}}}^{\mathrm{H}},\mathrm{t}\right)$ stems from the integral expression:

7 $${\mathbf{f}}^{\mathrm{H}}\left({\dot{\mathbf{f}}}^{\mathrm{H}},\mathrm{t}\right)={\int }_{0}^{\mathrm{t}}{\dot{\mathbf{f}}}^{\mathrm{H}}\left({\mathbf{f}}^{\mathrm{H}},\dot{{\varvec{\updelta}}}\right)\mathrm{d\tau }={\int }_{0}^{\mathrm{t}}{\mathbf{k}}^{\mathrm{H}}\left({\mathbf{f}}^{\mathrm{H}},\dot{{\varvec{\updelta}}}\right) \dot{{\varvec{\updelta}}}\left(\uptau \right)\mathrm{d\tau }$$

The nth hysteretic stiffness portion can account for a-symmetric yielding force, stiffness degradation, strength degradation, pinch effect, and slip effect. For the simplest case of symmetric yielding, no stiffness/strength degradations and no pinch/slip effect, for example, the hysteretic stiffness portion is expressed as:

8 $${\mathrm{k}}_{\mathrm{n}}^{\mathrm{H}}\left({\mathrm{f}}_{\mathrm{n}}^{\mathrm{H}}, {\dot{\updelta }}_{\mathrm{n}}\right)=\left(1-{\mathrm{a}}_{\mathrm{n}}\right){\mathrm{k}}_{\mathrm{n}}^{\mathrm{el}}\left[1-{\left(\frac{{\mathrm{f}}_{\mathrm{n}}^{\mathrm{H}}\left({\dot{\mathrm{f}}}_{\mathrm{n}}^{\mathrm{H}},\mathrm{t}\right)}{\left(1-{\mathrm{a}}_{\mathrm{n}}\right){\mathrm{f}}_{\mathrm{n}}^{\mathrm{yld}}}\right)}^{\nu }\left(0.5 \mathrm{sgn}\left({\dot{\updelta }}_{\mathrm{n}}\left(\mathrm{t}\right) {\mathrm{f}}_{\mathrm{n}}^{\mathrm{H}}\left({\dot{\mathrm{f}}}_{\mathrm{n}}^{\mathrm{H}},\mathrm{t}\right)\right)+0.5\right)\right]$$

where ${a}_{n}$ is the nth component of $\mathbf{a}$, ${\mathrm{f}}_{\mathrm{n}}^{\mathrm{H}}\left(\mathrm{t}\right)$ is the nth component of ${\mathbf{f}}^{\mathrm{H}}\left(\mathrm{t}\right)$, $\nu$ is a parameter controlling the transition from elastic to a plastic state, and ${f}_{n}^{yld}$ is the n^th^ story shear resisting force at first yield.

### Hysteretic SMA model

Graesser and Cozzarelli^28^ present one of the first hysteretic models that simulate the SMA strain rate under cyclic loading in its austenite and transition phases. Witting and Cozzarelli^29^ followed suit and elaborated on the model's parameters to improve the computational effort. Wilde et al.^27^ enhanced the model further by adding the third martensite phase and addressing the transition from the transition phase to the martensite phase. This paper employs the model of Wilde et al.^27^ to simulate the force SMARD force.

**Figure 2:**
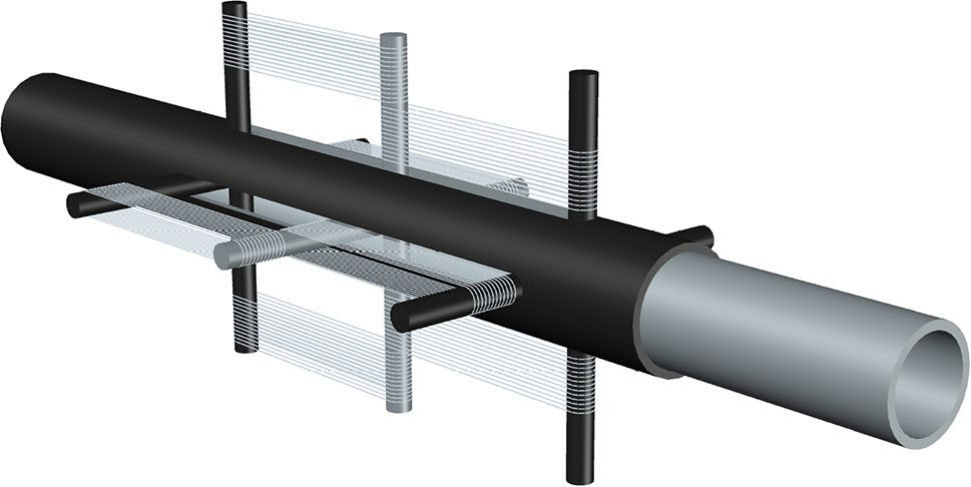
Shape-memory-alloy resisting device illustration.

**Figure 3:**
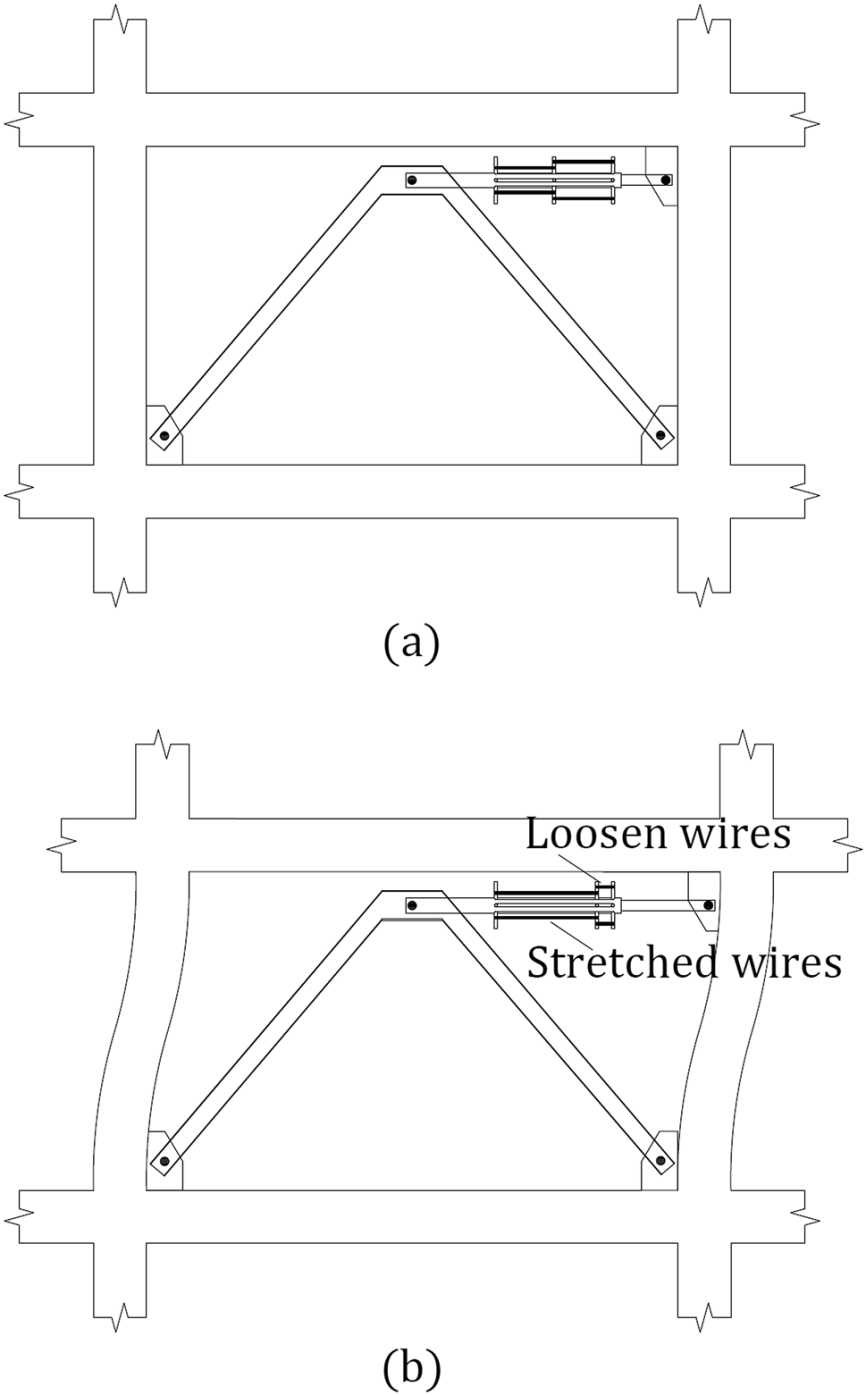
Shape-memory-alloy resisting device (**a**) installation scheme (**b**) under lateral deformation.

The SMARD installation scheme is depicted in Fig. 2. The SMARD consists of inner and outer steel tubes connected by SMA wire. Figure 3a exemplifies its installation in a frame system. As shown in Fig. 3b, when the frame undergoes lateral deformation, half of the wire loops experience tension and consequently introduce the SMARD force, while the other half loosens. In each lateral direction, a different group of wires experiences tension.

Similar to the model of Wang et al.^26^, the model of Wilde et al.^27^ addresses the SMARD force vector ${\mathbf{f}}^{\mathrm{SMA}}\left({\varvec{\updelta}},\dot{{\varvec{\updelta}}},\mathrm{t}\right)$ by referring to its rate ${\dot{\mathbf{f}}}^{\mathrm{SMA}}\left({\varvec{\updelta}},\dot{{\varvec{\updelta}}}\right)$. Hence:

9 $${\mathbf{f}}^{\mathrm{SMA}}\left({\varvec{\updelta}},\dot{{\varvec{\updelta}}},\mathrm{t}\right)={\int }_{0}^{\mathrm{t}}{\dot{\mathbf{f}}}^{\mathrm{SMA}}\left({\varvec{\updelta}},\dot{{\varvec{\updelta}}}\right)\mathrm{d\tau }$$

The model of ${\dot{\mathrm{f}}}_{\mathrm{n}}^{\mathrm{SMA}}\left(\mathrm{t}\right)$ is expressed by either referring to the axial strain or the SMA tangent stiffness:

10 $$\begin{array}{l}{\dot{\mathrm{f}}}_{\mathrm{n}}^{\mathrm{SMA}}\left({\updelta }_{\mathrm{n}},{\dot{\updelta }}_{\mathrm{n}}\right)={\mathrm{w}}_{\mathrm{n}}{\mathrm{A}}_{\mathrm{n}}^{\mathrm{SMA}}{\dot{\upsigma }}_{\mathrm{n}}^{\mathrm{SMA}}\left({\updelta }_{\mathrm{n}},{\dot{\updelta }}_{\mathrm{n}}\right)\\ \mathrm{or}\\ {\dot{\mathrm{f}}}_{\mathrm{n}}^{\mathrm{SMA}}\left({\updelta }_{\mathrm{n}},{\dot{\updelta }}_{\mathrm{n}}\right)={\mathrm{k}}_{\mathrm{n}}^{\mathrm{SMA}}\left({\updelta }_{\mathrm{n}},{\dot{\updelta }}_{\mathrm{n}}\right){\dot{\updelta }}_{\mathrm{n}}\left(\mathrm{t}\right)\end{array}$$

where ${w}_{n}$ is the number of SMA wires that undergo tension in the *n*th story (and the design variable of this paper), ${A}_{n}^{SMA}$ is a single wire effective area, ${\sigma }_{n}^{SMA}\left({\delta }_{n},{\dot{\delta }}_{n}\right)$ is the axial stress, and ${k}_{n}^{SMA}\left({\delta }_{n},{\dot{\delta }}_{n}\right)$ is the nth story's SMARDs' tangent stiffness. All wires' area, length, and material are considered identical in the n^th^ story. According to the modified constitutive law for SMA material as expressed in Wilde et al.^27^, the axial stress rate ${\dot{\sigma }}_{n}^{SMA}\left(\tau \right)$ is given by:

11 $$\begin{aligned}{}&{\dot{\upsigma }}_{\mathrm{n}}^{\mathrm{SMA}}\left({\updelta }_{\mathrm{n}},{\dot{\updelta }}_{\mathrm{n}}\right)={\mathrm{E}}_{\mathrm{n}}^{\mathrm{SMA}}\left[{\dot{\upepsilon }}_{\mathrm{n}}^{\mathrm{SMA}}\left(\mathrm{t}\right)-\left|{\dot{\upepsilon }}_{\mathrm{n}}^{\mathrm{SMA}}\left(\mathrm{t}\right)\right|{\left({\uprho }_{\mathrm{n}}^{\mathrm{SMA}}\left(\mathrm{t}\right)\right)}^{{\upgamma }_{1}}\right]{\mathrm{u}}_{\mathrm{I}}+{\mathrm{E}}_{\mathrm{n}}^{\mathrm{m},\mathrm{SMA}}{\dot{\upepsilon }}_{\mathrm{n}}^{\mathrm{SMA}}\left(\mathrm{t}\right){\mathrm{u}}_{\mathrm{II}}\\&\quad +\dots \left(3{\uptheta }_{1}{\dot{\upepsilon }}_{\mathrm{n}}^{\mathrm{SMA}}\left(\mathrm{t}\right){\left({\upepsilon }_{\mathrm{n}}^{\mathrm{SMA}}\right)}^{2}+2{\uptheta }_{2}{\dot{\upepsilon }}_{\mathrm{n}}^{\mathrm{SMA}}\left(\mathrm{t}\right)\left|{\upepsilon }_{\mathrm{n}}^{\mathrm{SMA}}\right|+{\uptheta }_{3}{\dot{\upepsilon }}_{\mathrm{n}}^{\mathrm{SMA}}\left(\mathrm{t}\right)\right){\mathrm{u}}_{\mathrm{III}}\\ & \mathrm{and}:\\ &{\mathrm{u}}_{\mathrm{I}}=1-{\mathrm{u}}_{\mathrm{II}}\left({\updelta }_{\mathrm{n}}\right)-{\mathrm{u}}_{\mathrm{III}}\left({\updelta }_{\mathrm{n}},{\dot{\updelta }}_{\mathrm{n}}\right)\\ &{\mathrm{u}}_{\mathrm{II}}=0.5\left(1+\mathrm{erf}\left\{\upbeta \left(\left|{\upepsilon }_{\mathrm{n}}^{\mathrm{SMA}}\left(\mathrm{t}\right)\right|-{\upepsilon }_{\mathrm{n}}^{\mathrm{m},\mathrm{SMA}}\right)\right\}\right)\\ &{\mathrm{u}}_{\mathrm{III}}=0.5\left[1+0.5\mathrm{erf}\left\{\upbeta {\upepsilon }_{\mathrm{n}}^{\mathrm{SMA}}\left(\mathrm{t}\right){\dot{\upepsilon }}_{\mathrm{n}}^{\mathrm{SMA}}\left(\mathrm{t}\right)\right\}\right.\left(\mathrm{erf}\left\{\upbeta \left(\left|{\upepsilon }_{\mathrm{n}}^{\mathrm{SMA}}\left(\mathrm{t}\right)\right|-{\upepsilon }_{\mathrm{n}}^{1,\mathrm{SMA}}\right)\right\}\right.\\ &\quad \left.\left.+\dots +\mathrm{erf}\left\{\upbeta \left({\upepsilon }_{\mathrm{n}}^{\mathrm{m},\mathrm{SMA}}-\left|{\upepsilon }_{\mathrm{n}}^{\mathrm{SMA}}\left(\mathrm{t}\right)\right|\right)\right\}\right)\right]\end{aligned}$$

12 $${\upepsilon }_{\mathrm{n}}^{\mathrm{SMA}}\left(\mathrm{t}\right)={\updelta }_{\mathrm{n}}\left(\mathrm{t}\right)$$

13 $${\dot{\upepsilon }}_{\mathrm{n}}^{\mathrm{SMA}}\left(\mathrm{t}\right)={\dot{\updelta }}_{\mathrm{n}}\left(\mathrm{t}\right)$$

where ${E}_{n}^{SMA}$ is the *n*^th^ story SMA modulus of elasticity in the austenite phase, ${E}_{n}^{m,SMA}$ is the modulus of elasticity in the martensite phase, ${\gamma }_{1}$ is a model parameter controlling the transition between austenite and transition phases, ${L}_{n}^{SMA}$ is the initial length of the n^th^ story SMA wires, ${\epsilon }_{n}^{SMA}\left(t\right)$ is the axial strain, ${\dot{\epsilon }}_{n}^{SMA}\left(t\right)$ is the strain rate, ${\epsilon }_{n}^{1,SMA}$ is the axial strain at the beginning of the martensite phase transition, ${\epsilon }_{n}^{m,SMA}$ is where the martensite transition ends and the material is in the martensite phase, and $\beta$ is a large positive number providing an almost immediate change from − 1 to 1 and vice-versa. The constants ${\theta }_{1}$, ${\theta }_{2}$, and ${\theta }_{3}$ define the curvature of the martensite transition. The function ${\rho }_{n}^{SMA}\left(t\right)$ is an index function describing the relationship between the applied axial stress (axial stress minus the back stress) divided by the critical stress:

14 $${\uprho }_{\mathrm{n}}^{\mathrm{SMA}}\left(\mathrm{t}\right)=\frac{{\upsigma }_{\mathrm{n}}^{\mathrm{SMA}}\left({\updelta }_{\mathrm{n}},{\dot{\updelta }}_{\mathrm{n}}\right)-{\upsigma }_{\mathrm{n}}^{\mathrm{SMA},\mathrm{back}}\left({\updelta }_{\mathrm{n}},{\dot{\updelta }}_{\mathrm{n}}\right)}{{\upsigma }_{\mathrm{n}}^{\mathrm{SMA},\mathrm{crc}}}$$

In Eq. (14), ${\sigma }_{n}^{SMA,back}$ is the back-stress and ${\sigma }_{n}^{SMA,crc}$ is the critical-stress, which are given by:

15 $$\begin{array}{l}{\upsigma }_{\mathrm{n}}^{\mathrm{SMA},\mathrm{back}}\left({\updelta }_{\mathrm{n}},{\dot{\updelta }}_{\mathrm{n}}\right)={\mathrm{E}}_{\mathrm{n}}^{\mathrm{SMA}}{\mathrm{\alpha }}_{\mathrm{n}}^{\mathrm{SMA}}\left({\upepsilon }_{\mathrm{n}}^{\mathrm{SMA},\mathrm{in}}\left(\mathrm{t}\right)+{\mathrm{f}}^{\mathrm{T}}{\left|{\upepsilon }_{\mathrm{n}}^{\mathrm{SMA}}\left(\mathrm{t}\right)\right|}^{{\upgamma }_{2}}\mathrm{erf}\left(\upbeta {\upepsilon }_{\mathrm{n}}^{\mathrm{SMA}}\left(\mathrm{t}\right)\right){\mathrm{u}}_{\mathrm{IV}}\right)\\ \mathrm{and}:\\ {\mathrm{u}}_{\mathrm{IV}}=0.5\left(1+\mathrm{erf}\left\{\upbeta \left(-{\upepsilon }_{\mathrm{n}}^{\mathrm{SMA}}\left(\mathrm{t}\right){\dot{\upepsilon }}_{\mathrm{n}}^{\mathrm{SMA}}\left(\mathrm{t}\right)\right)\right\}\right)\end{array}$$

16 $${\upsigma }_{\mathrm{n}}^{\mathrm{SMA},\mathrm{crc}}={\upsigma }_{\mathrm{n}}^{\mathrm{SMA},\mathrm{yld}}-{\mathrm{f}}^{\mathrm{T}}{\mathrm{E}}_{\mathrm{n}}^{\mathrm{SMA}}{\mathrm{\alpha }}_{\mathrm{n}}^{\mathrm{SMA}}{\left(1+{\mathrm{\alpha }}_{\mathrm{n}}^{\mathrm{SMA}}\right)}^{\frac{1}{{\upgamma }_{1}}}$$

where the stress ${\sigma }_{n}^{SMA,yld}$ represents the SMA material yield stress (i.e., the stress of the transition between the austenite and transition phases), ${f}^{T}$ is a parameter that controls the back-stress reduction portion, the coefficient ${\alpha }_{n}^{SMA}$ represents a relationship between the elastic and inelastic moduli, given by:

17 $${\mathrm{\alpha }}_{\mathrm{n}}^{\mathrm{SMA}}=\frac{{\mathrm{E}}_{\mathrm{n}}^{\mathrm{SMA},\mathrm{in}}}{{\mathrm{E}}_{\mathrm{n}}^{\mathrm{SMA}}+{\mathrm{E}}_{\mathrm{n}}^{\mathrm{SMA},\mathrm{in}}}$$

And ${\epsilon }_{n}^{SMA,in}\left(t\right)$ is the inelastic strain portion:

18 $${\upepsilon }_{\mathrm{n}}^{\mathrm{SMA},\mathrm{in}}\left(\mathrm{t}\right)={\upepsilon }_{\mathrm{n}}^{\mathrm{SMA}}\left(\mathrm{t}\right)-\frac{{\upsigma }_{\mathrm{n}}^{\mathrm{SMA}}\left(\mathrm{t}\right)}{{\mathrm{E}}_{\mathrm{n}}^{\mathrm{SMA}}}$$

Substituting the strain rate expression of Eq. (13) into Eq. (11) and multiplying both sides by ${w}_{n}{A}_{n}^{SMA}$ yields the expression for the tangent stiffness of the SMARD:

19 $$\begin{array}{l}{\mathrm{k}}_{\mathrm{n}}^{\mathrm{SMA}}\left({\updelta }_{\mathrm{n}},{\dot{\updelta }}_{\mathrm{n}}\right)={\mathrm{w}}_{\mathrm{n}}\frac{{\mathrm{E}}_{\mathrm{n}}^{\mathrm{SMA}}{\mathrm{A}}_{\mathrm{n}}^{\mathrm{SMA}}}{{\mathrm{L}}_{\mathrm{n}}^{\mathrm{SMA}}}{\uppsi }_{\mathrm{n}}\left({\upepsilon }_{\mathrm{n}}^{\mathrm{SMA}},{\dot{\updelta }}_{\mathrm{n}}\right)\\ \mathrm{and}:\\ \begin{array}{l}{\uppsi }_{\mathrm{n}}\left({\updelta }_{\mathrm{n}},{\dot{\updelta }}_{\mathrm{n}}\right)=\left[1-\frac{{\mathrm{L}}_{\mathrm{n}}^{\mathrm{SMA}}}{{\dot{\updelta }}_{\mathrm{n}}\left(\mathrm{t}\right)}{\left({\uprho }_{\mathrm{n}}^{\mathrm{SMA}}\left(\mathrm{t}\right)\right)}^{{\upgamma }_{1}}\mathrm{sgn}\left({\dot{\updelta }}_{\mathrm{n}}\left(\mathrm{t}\right)\right)\right]{\mathrm{u}}_{\mathrm{I}}\left(\mathrm{t}\right)+{\mathrm{E}}_{\mathrm{n}}^{\mathrm{m},\mathrm{SMA}}{\mathrm{u}}_{\mathrm{II}}\left(\mathrm{t}\right)\\ +\dots \left(3{\uptheta }_{1}{\left({\upepsilon }_{\mathrm{n}}^{\mathrm{SMA}}\right)}^{2}+2{\uptheta }_{2}\left|{\upepsilon }_{\mathrm{n}}^{\mathrm{SMA}}\right|+{\uptheta }_{3}\right){\mathrm{u}}_{\mathrm{III}}\left(\mathrm{t}\right)\end{array}\end{array}$$

So that ${\uppsi }_{\mathrm{n}}\left({\updelta }_{\mathrm{n}},{\dot{\updelta }}_{\mathrm{n}}\right)$ is a function that governs the cyclic behavior of the SMA wires in the nth story. Given the nth story tangent stiffness definition, the diagonal tangent SMA stiffness matrix ${\mathbf{k}}^{\mathrm{SMA}}\left({\varvec{\updelta}},\dot{{\varvec{\updelta}}}\right)$ is composed of the stories' tangent stiffnesses as follows:

20 $$\begin{array}{ccc}{\dot{\mathbf{f}}}^{\mathrm{SMA}}\left({\varvec{\updelta}},\dot{{\varvec{\updelta}}}\right)={\mathbf{k}}^{\mathrm{SMA}}\left({\varvec{\updelta}},\dot{{\varvec{\updelta}}}\right)\dot{{\varvec{\updelta}}}\left(\mathrm{t}\right)& \leftrightarrow & {\mathbf{k}}^{\mathrm{SMA}}\left({\varvec{\updelta}},\dot{{\varvec{\updelta}}}\right)=\left[\begin{array}{ccc}{\mathrm{k}}_{1}^{\mathrm{SMA}}\left({\updelta }_{1},{\dot{\updelta }}_{1}\right)& & \\ & \ddots & \\ & & {\mathrm{k}}_{\mathrm{N}}^{\mathrm{SMA}}\left({\updelta }_{\mathrm{N}},{\dot{\updelta }}_{\mathrm{N}}\right)\end{array}\right]\end{array}$$

Since this paper deals with optimizing the number of SMA wires allocated to the stories', the wires quantity is extracted from the expression of ${\mathbf{k}}^{\mathrm{SMA}}\left({\varvec{\updelta}},\dot{{\varvec{\updelta}}}\right)$, so that:

21 $${\dot{\mathbf{f}}}^{\mathrm{SMA}}\left({\varvec{\updelta}},\dot{{\varvec{\updelta}}}\right)=\mathbf{W}{\mathbf{k}}^{\mathrm{w}0}\left({\varvec{\updelta}},\dot{{\varvec{\updelta}}}\right)\dot{{\varvec{\updelta}}}\left(\mathrm{t}\right)$$

so that:

22 $$\begin{array}{ccc}\mathbf{W}=\mathrm{diag}\left\{\mathbf{w}\right\}& \leftrightarrow & \mathbf{w}=\left[\begin{array}{c}{\mathrm{w}}_{1}\\ \vdots \\ {\mathrm{w}}_{\mathrm{N}}\end{array}\right]\end{array}$$

23 $${\mathbf{k}}^{\mathrm{w}0}\left({{\varvec{\upepsilon}}}^{\mathrm{SMA}},\dot{{\varvec{\updelta}}}\right)=\left[\begin{array}{ccc}\frac{{\mathrm{E}}_{1}^{\mathrm{SMA}}{\mathrm{A}}_{1}^{\mathrm{SMA}}}{{\mathrm{L}}_{1}^{\mathrm{SMA}}}& & \\ & \ddots & \\ & & \frac{{\mathrm{E}}_{\mathrm{N}}^{\mathrm{SMA}}{\mathrm{A}}_{\mathrm{N}}^{\mathrm{SMA}}}{{\mathrm{L}}_{\mathrm{N}}^{\mathrm{SMA}}}\end{array}\right]\left[\begin{array}{ccc}{\uppsi }_{1}\left({\updelta }_{1},{\dot{\updelta }}_{1}\right)& & \\ & \ddots & \\ & & {\uppsi }_{\mathrm{N}}\left({\updelta }_{\mathrm{N}},{\dot{\updelta }}_{\mathrm{N}}\right)\end{array}\right]$$

where "$\mathrm{diag}\left\{\right\}$" turns a vector into a diagonal matrix. In this form, the vector $\mathbf{w}$ consists of the design variables, $\mathbf{W}$ is a diagonal matrix whose components are the wires quantity in each story, and ${\mathbf{k}}^{\mathrm{w}0}\left({{\varvec{\upepsilon}}}^{\mathrm{SMA}},\dot{{\varvec{\updelta}}}\right)$ represents the SMA material's hysteretic behavior. Equation (21) is adopted and implemented in the following problem formulation.

## Control gain optimization

### Problem definition

This paper aims to optimize the system's performance by reducing the inelastic control state response subject to dynamic equilibrium and the minimum/maximum number of SMA wires assigned. Define the following control gain optimization problem:

24 $$\begin{array}{ll}\underset{\mathbf{w}}{\mathrm{minimize}}& \left\{\mathrm{J}\left(\mathbf{w}\right)={\int }_{0}^{{\mathrm{t}}_{\mathrm{f}}}{\mathbf{z}\left(\mathrm{t}\right)}^{\mathrm{T}}\mathbf{Q}\mathbf{z}\left(\mathrm{t}\right)\mathrm{dt}\right\}\\ \mathrm{subject \,\, to}& \dot{\mathbf{z}}\left(\mathrm{t}\right)=\mathbf{A}\left(\mathbf{z}\left(\mathrm{t}\right)\right)\mathbf{z}\left(\mathrm{t}\right)+\mathbf{B}\left(\mathrm{t}\right)\left(-\mathbf{m}{\mathbf{e}}_{1}{\mathrm{a}}_{\mathrm{g}}\left(\mathrm{t}\right)+\mathbf{u}\left(\mathbf{w},\mathbf{z}\left(\mathrm{t}\right)\right)\right)\\ & \mathbf{z}\left(0\right)=0\\ & \mathbf{w}-{\mathbf{w}}^{\mathrm{max}}\le 0\\ & {\mathbf{w}}^{\mathrm{min}}-\mathbf{w}\le 0\end{array}$$

In Eq. (24), the objective function is subject to the state-space representation, and the maximum number of wires allowed dictate by the vectors ${\mathbf{w}}^{\mathrm{max}}$ and ${\mathbf{w}}^{\mathrm{min}}$. The state-vector of the inelastic system $\mathbf{z}\left(\mathrm{t}\right)$ is defined as:

25 $$\begin{array}{cccc}\mathbf{z}\left(\mathrm{t}\right)=\left[\begin{array}{c}\mathbf{y}\left(\mathrm{t}\right)\\ \dot{\mathbf{y}}\left(\mathrm{t}\right)\end{array}\right]& \leftrightarrow & \mathbf{y}\left(\mathrm{t}\right)=\left[\begin{array}{c}{\varvec{\updelta}}\left(\mathrm{t}\right)\\ {\int }_{0}^{\mathrm{t}}{\mathbf{f}}^{\mathrm{H}}\left({\dot{\mathbf{f}}}^{\mathrm{H}},\uptau \right)\mathrm{d\tau }\end{array}\right],& \dot{\mathbf{y}}\left(\mathrm{t}\right)=\left[\begin{array}{c}\dot{{\varvec{\updelta}}}\left(\mathrm{t}\right)\\ {\mathbf{f}}^{\mathrm{H}}\left({\dot{\mathbf{f}}}^{\mathrm{H}},\mathrm{t}\right)\end{array}\right]\end{array}$$

So that the corresponding non-autonomous state matrix is given by:

26 $$\mathbf{A}\left(\mathbf{z}\left(\mathrm{t}\right)\right)=\left[\begin{array}{cccc}0& 0& \mathbf{I}& 0\\ 0& 0& 0& \mathbf{I}\\ -{\mathbf{m}}^{-1}\left(\mathbf{a} {\mathbf{k}}^{\mathrm{el}}\right)& 0& -{\mathbf{m}}^{-1}\mathbf{c}& -{\mathbf{m}}^{-1}\\ 0& 0& {\mathbf{k}}^{\mathrm{H}}\left({\mathbf{f}}^{\mathrm{H}},\dot{{\varvec{\updelta}}}\right)& 0\end{array}\right]$$

where $\mathbf{I}$ is the *N* × *N* identity matrix, and $0$ is the *N* × *N* zero matrix. The input $\mathbf{u}\left(\mathbf{w},\mathbf{z}\left(\mathrm{t}\right)\right)$ is the control force and is given by the active control gain $\mathbf{G}\left(\mathbf{w},\mathbf{z}\left(\mathrm{t}\right)\right)$ as follows:

27 $$\begin{array}{ccc}\mathbf{u}\left(\mathbf{w},\mathbf{z}\left(\mathrm{t}\right)\right)=-{\int }_{0}^{\mathrm{t}}\mathbf{G}\left(\mathbf{w},\mathbf{z}\left(\mathrm{t}\right)\right)\mathbf{z}\left(\uptau \right)\mathrm{d\tau }& \leftrightarrow & \mathbf{G}\left(\mathbf{w},\mathbf{z}\left(\mathrm{t}\right)\right)=\left[\begin{array}{cccc}0& 0& \mathbf{w}{\mathbf{k}}^{\mathrm{w}0}\left({\varvec{\updelta}}\left(\mathrm{t}\right),\dot{{\varvec{\updelta}}}\left(\mathrm{t}\right)\right)& 0\end{array}\right]\end{array}$$

where the input-to-state matrix is given by:

28 $$\mathbf{B}=\left[\begin{array}{c}0\\ 0\\ {\mathbf{m}}^{-1}\\ 0\end{array}\right]$$

Equation (25) defines a unique state vector $\mathbf{z}\left(\mathrm{t}\right)$ consisting of the interstory drifts vector and the time integral of the hysteretic portion of the resisting shear force vector. The proposed state vector yields an idealized state matrix and state-space representation for the system's inelasticity. The objective function looks to minimize the components of $\mathbf{z}\left(\mathrm{t}\right)$ whose relative importance is defined by the optimal weighting matrix $\mathbf{Q}$. The numerical example examines the comparative effect between the deformation, velocity, and the force related components of $\mathbf{Q}$.

### Optimization strategy

The control gain optimization problem of Eq. (24) is solved using Pontryagin's minimum principle for a finite time problem so as to calculate the optimal SMA wires while taking into account the minimum/maximum design limitations. Define the Hamiltonian as:

29 $$\mathcal{H}\left(\mathrm{t},\mathbf{z},\mathbf{w},{\varvec{\uplambda}}\right)={\mathbf{z}}^{\mathrm{T}}\mathbf{Q}\mathbf{z}+{{\varvec{\uplambda}}}^{\mathrm{T}}\left[\mathbf{A}\left(\mathbf{z}\right)\mathbf{z}+\mathbf{B}\left(-\mathbf{m}{\mathbf{e}}_{1}{\mathrm{a}}_{\mathrm{g}}\left(\mathrm{t}\right)+\mathbf{u}\left(\mathbf{w},\mathbf{z}\right)\right)\right]$$

where ${\varvec{\uplambda}}\left(\mathrm{t}\right)$ is a *4 N*-dimensional vector correlated to the time-varying Lagrange multipliers. The Lagrangian of Eq. (24) is given by:

30 $$\begin{array}{l}\mathcal{L}\left(\mathbf{z},\mathbf{w},{\varvec{\uplambda}},{\varvec{\upkappa}},{\varvec{\upmu}}\right)={\int }_{0}^{{\mathrm{t}}_{\mathrm{f}}}{\mathbf{z}\left(\mathrm{t}\right)}^{\mathrm{T}}\mathbf{Q}\mathbf{z}\left(\mathrm{t}\right)+{{\varvec{\uplambda}}}^{\mathrm{T}}\left(\mathrm{t}\right)\left[\mathbf{A}\left(\mathbf{z}\left(\mathrm{t}\right)\right)\mathbf{z}\left(\mathrm{t}\right)+\mathbf{B}\left(-\mathbf{m}{\mathbf{e}}_{1}{\mathrm{a}}_{\mathrm{g}}\left(\mathrm{t}\right)+\mathbf{u}\left(\mathbf{w},\mathbf{z}\left(\mathrm{t}\right)\right)\right)-\dot{\mathbf{z}}\left(\mathrm{t}\right)\right]\mathrm{dt}+\dots \\ {{\varvec{\upkappa}}}^{\mathrm{T}}\left(\mathbf{z}\left(0\right)-0\right)+{{\varvec{\upmu}}}^{\mathrm{T}}\left[\begin{array}{c}\mathbf{w}-{\mathbf{w}}^{\mathrm{max}}\\ {\mathbf{w}}^{\mathrm{min}}-\mathbf{w}\end{array}\right]\end{array}$$

Here, the Lagrangian expression comprises the objective function combined with the state-space representation multiplied by the time-varying Lagrange multipliers, coefficients governing the zero initial state ${\varvec{\upkappa}}$, and the inequality constraint multiplied by the KKT multipliers vector ${\varvec{\upmu}}$. Placing the Hamiltonian of Eq. (29) in the Lagrangian expression provides:

31 $$\mathcal{L}\left(\mathbf{z},\mathbf{w},{\varvec{\uplambda}},{\varvec{\upkappa}},{\varvec{\upmu}}\right)={\int }_{0}^{{\mathrm{t}}_{\mathrm{f}}}\mathcal{H}\left(\mathrm{t},\mathbf{z}\left(\mathrm{t}\right),\mathbf{w},{\varvec{\uplambda}}\left(\mathrm{t}\right)\right)-{{\varvec{\uplambda}}}^{\mathrm{T}}\left(\mathrm{t}\right)\dot{\mathbf{z}}\left(\mathrm{t}\right)\mathrm{dt}+{{\varvec{\upkappa}}}^{\mathbf{T}}\left(\mathbf{z}\left(0\right)-0\right)+{{\varvec{\upmu}}}^{\mathrm{T}}\left[\begin{array}{c}\mathbf{w}-{\mathbf{w}}^{\mathrm{max}}\\ {\mathbf{w}}^{\mathrm{min}}-\mathbf{w}\end{array}\right]$$

One of the most basic methods for solving (unconstrained) optimization problems is the gradient method or method of steepest descent. The idea of this method is to iteratively follow the direction of the steepest descent of a given objective function at a current iterate to converge to a stationary point. The gradient method is usually combined with a line-search strategy like Armijo's rule to achieve convergence to a fixed point from arbitrary starting points.

We follow a shooting (or reduction) approach and assume that $\mathbf{z}\left(\mathrm{t}\right)$ is determined through a given $\mathbf{w}$—defining the parameter-to-state mapping:

32 $${\mathbf{w}} \mapsto {\mathbf{z}}\left( {{\text{t}},{\mathbf{w}}} \right)$$

Also, we assume the following:

(i) The initial value problem $\dot{\mathbf{z}}\left(\mathrm{t}\right)=\mathbf{A}\left(\mathbf{z}\left(\mathrm{t}\right)\right)\mathbf{z}\left(\mathrm{t}\right)+\mathbf{B}\left(-\mathbf{m}{\mathbf{e}}_{1}{\mathrm{a}}_{\mathrm{g}}\left(\mathrm{t}\right)+\mathbf{u}\left(\mathbf{w},\mathbf{z}\left(\mathrm{t}\right)\right)\right)$, $\mathbf{z}\left(0\right)=0$, possesses a unique solution for every $\mathbf{w}$.
(ii) The mapping ${\mathbf{w}} \mapsto {\mathbf{z}}\left( {{\text{t}},{\mathbf{w}}} \right)$ is continuously Frechet-differentiable (derivative defined on normed spaces).

In order to compute the gradient of $J$ at some $\widehat{\mathbf{w}}$ and of trajectory $\widehat{\mathbf{z}}\left(\mathrm{t}\right)=\mathbf{z}\left(\mathrm{t},\widehat{\mathbf{w}}\right)$ we consider the auxiliary functional:

33 $$\begin{array}{l}\widetilde{\mathrm{J}}\left(\widehat{\mathbf{w}}\right)=\mathrm{J}\left(\widehat{\mathbf{w}}\right)+{\int }_{0}^{{\mathrm{t}}_{\mathrm{f}}}{{\varvec{\uplambda}}}^{\mathrm{T}}\left(\mathrm{t}\right)\left[\mathbf{A}\left(\widehat{\mathbf{z}}\left(\mathrm{t}\right)\right)\widehat{\mathbf{z}}\left(\mathrm{t}\right)+\mathbf{B}\left(-\mathbf{m}{\mathbf{e}}_{1}{\mathrm{a}}_{\mathrm{g}}\left(\mathrm{t}\right)+\mathbf{u}\left(\widehat{\mathbf{w}},\widehat{\mathbf{z}}\left(\mathrm{t}\right)\right)\right)-\dot{\widehat{\mathbf{z}}}\left(\mathrm{t}\right)\right]\mathrm{dt}=\dots \\ {\int }_{0}^{{\mathrm{t}}_{\mathrm{f}}}\mathcal{H}\left(\mathrm{t},\widehat{\mathbf{z}}\left(\mathrm{t}\right),\widehat{\mathbf{w}},{\varvec{\uplambda}}\left(\mathrm{t}\right)\right)-{{\varvec{\uplambda}}}^{\mathrm{T}}\left(\mathrm{t}\right)\dot{\widehat{\mathbf{z}}}\left(\mathrm{t}\right)\mathrm{dt}\end{array}$$

Using partial integration, we get:

34 $$\widetilde{\mathrm{J}}\left(\widehat{\mathbf{w}}\right)=-{\left[{{\varvec{\uplambda}}}^{\mathrm{T}}\left(\mathrm{t}\right)\widehat{\mathbf{z}}\left(\mathrm{t}\right)\right]}_{0}^{{\mathrm{t}}_{\mathrm{f}}}+{\int }_{0}^{{\mathrm{t}}_{\mathrm{f}}}\mathcal{H}\left(\mathrm{t},\widehat{\mathbf{z}}\left(\mathrm{t}\right),\widehat{\mathbf{w}},{\varvec{\uplambda}}\left(\mathrm{t}\right)\right)+{\dot{{\varvec{\uplambda}}}}^{\mathrm{T}}\left(\mathrm{t}\right)\widehat{\mathbf{z}}\left(\mathrm{t}\right)\mathrm{dt}$$

Denote the sensitivity of $\widehat{\mathbf{z}}\left(\mathrm{t}\right)$ in $\widehat{\mathbf{w}}$ as:

35 $$\mathbf{S}\left(\mathrm{t}\right)={\mathbf{z}}_{\widehat{\mathbf{w}}}\left(\mathrm{t},\widehat{\mathbf{w}}\right)$$

Formal differentiation of $\widetilde{\mathrm{J}}\left(\mathbf{w}\right)$ at $\mathbf{w}$ in the direction $\widehat{\mathbf{w}}$ and exploitation of $\mathbf{S}\left(0\right)=0$ (since $\mathbf{z}\left(0\right)$ is fixed), yields the term:

36 $${\widetilde{\mathrm{J}}}_{\widehat{\mathbf{w}}}\left(\mathbf{w}\right)=-{{\varvec{\uplambda}}}^{\mathrm{T}}\left({\mathrm{t}}_{\mathrm{f}}\right)\mathbf{S}\left({\mathrm{t}}_{\mathrm{f}}\right)+{\int }_{0}^{{\mathrm{t}}_{\mathrm{f}}}{\left({\nabla }_{\mathbf{z}}\mathcal{H}\left(\mathrm{t}\right)+{\dot{{\varvec{\uplambda}}}}^{\mathrm{T}}\left(\mathrm{t}\right)\right)}^{\mathrm{T}}\mathbf{S}\left(\mathrm{t}\right)+{\nabla }_{\mathbf{w}}\mathcal{H}\left(\mathrm{t}\right)\mathbf{w}\mathrm{dt}$$

As the sensitivity $\mathbf{S}\left(\mathrm{t}\right)$ is expensive to compute, ${\varvec{\uplambda}}\left(\mathrm{t}\right)$ is chosen in such a way that the corresponding terms are eliminated.

The necessary conditions for optimality are derived according to Theorem 2.3.24 in Chapter 2 in the book of Gerdts 2011^30^. That is:

37 $$\dot{{\varvec{\uplambda}}}\left(\mathrm{t}\right)=-{\nabla }_{\mathbf{z}}\mathcal{H}\left(\mathrm{t},\mathbf{z}\left(\mathrm{t}\right),\mathbf{w},{\varvec{\uplambda}}\left(\mathrm{t}\right)\right)$$

38 $${\varvec{\uplambda}}\left({\mathrm{t}}_{\mathrm{f}}\right)=0$$

where Eqs. (37) and (38) are the adjoint and transversality optimality conditions. That reduces ${\widetilde{\mathrm{J}}}_{\widehat{\mathbf{w}}}\left(\widehat{\mathbf{w}}\right)$ to:

39 $${\widetilde{\mathrm{J}}}_{\widehat{\mathbf{w}}}\left(\mathbf{w}\right)={\int }_{0}^{{\mathrm{t}}_{\mathrm{f}}}{\nabla }_{\mathbf{w}}\mathcal{H}\left(\mathrm{t}\right)\mathbf{w}\mathrm{dt}={\int }_{0}^{{\mathrm{t}}_{\mathrm{f}}}{\nabla }_{\mathbf{w}}\mathcal{H}\left(\mathrm{t}\right)\mathrm{dt}\mathbf{w}$$

Define:

40 $${\varvec{\upeta}}\left(\mathrm{t}\right)={\int }_{\mathrm{t}}^{{\mathrm{t}}_{\mathrm{f}}}{\nabla }_{\mathbf{w}}\mathcal{H}\left(\uptau \right)\mathrm{d\tau }$$

Added with the adjoint and transversality conditions:

41 $$\dot{{\varvec{\upeta}}}\left(\mathrm{t}\right)=-{\nabla }_{\mathbf{w}}\mathcal{H}\left(\mathrm{t},\mathbf{z}\left(\mathrm{t}\right),\mathbf{w},{\varvec{\uplambda}}\left(\mathrm{t}\right)\right)$$

42 $${\varvec{\upeta}}\left({\mathrm{t}}_{\mathrm{f}}\right)=0$$

Then, substituting Eq. (40) into Eq. (39) yields:

43 $${\widetilde{\mathrm{J}}}_{\widehat{\mathbf{w}}}\left(\mathbf{w}\right)={{\varvec{\upeta}}\left(0\right)}^{\mathrm{T}}\mathbf{w}$$

And, thus, ${\varvec{\upeta}}\left(0\right)$ is the gradient of $\widetilde{\mathrm{J}}\left(\widehat{\mathbf{w}}\right)$ at $\widehat{\mathbf{w}}$. Since $\widetilde{\mathrm{J}}\left(\mathbf{w}\right)=\mathrm{J}\left(\mathbf{w}\right)$ for every $\mathbf{w}$, we have proven:

#### Theorem

*Let* $\mathbf{w}$ *be given, let the assumptions (i) and (ii) be satisfied, and let* ${\varvec{\uplambda}}\left(\mathrm{t}\right)$ *and* ${\varvec{\upeta}}\left(0\right)$ *satisfy the adjoint equations. Then,* ${\nabla }_{\mathbf{w}}\mathrm{J}\left(\mathbf{w}\right)={\varvec{\upeta}}\left(0\right)$.

The Hamiltonian's gradients in $\mathbf{z}\left(\mathrm{t}\right)$ and $\mathbf{w}$ are given by:

44 $${\nabla }_{\mathbf{z}}\mathcal{H}\left(\mathrm{t},\mathbf{z}\left(\mathrm{t}\right),\mathbf{w},{\varvec{\uplambda}}\left(\mathrm{t}\right)\right)=2\mathbf{Q}\mathbf{z}+\left(\mathbf{A}\left(\mathbf{z}\left(\mathrm{t}\right)\right)+{\nabla }_{\mathbf{z}}\mathbf{A}\left(\mathbf{z}\left(\mathrm{t}\right)\right)\mathbf{z}\left(\mathrm{t}\right)-\mathbf{B}{\int }_{0}^{\mathrm{t}}\mathbf{G}\left(\mathbf{w},\mathbf{z}\left(\uptau \right)\right)\mathrm{d\tau }\right){\varvec{\uplambda}}$$

45 $${\nabla }_{\mathbf{w}}\mathcal{H}\left(\mathrm{t},\widehat{\mathbf{z}}\left(\mathrm{t}\right),\widehat{\mathbf{w}},{\varvec{\uplambda}}\left(\mathrm{t}\right)\right)=-\mathrm{diag}\left\{{\int }_{0}^{\mathrm{t}}\left[\begin{array}{cccc}0& 0& {\mathbf{k}}^{\mathrm{w}0}\left({\varvec{\updelta}}\left(\uptau \right),\dot{{\varvec{\updelta}}}\left(\uptau \right)\right)& 0\end{array}\right]\mathbf{z}\left(\uptau \right)\mathrm{d\tau }\right\}{\mathbf{B}}^{\mathrm{T}}{\varvec{\uplambda}}\left(\mathrm{t}\right)$$

So that ${\nabla }_{\mathbf{z}}\mathbf{A}\left(\mathbf{z}\left(\mathrm{t}\right)\right)\mathbf{z}\left(\mathrm{t}\right)$ is calculated in a discrete-time manner, as explained in the subsequent section.

## Numeric evaluation of λ(t) and η(t)

The calculation of ${\varvec{\uplambda}}\left(\mathrm{t}\right)$ and ${\varvec{\upeta}}\left(\mathrm{t}\right)$ requires solving $\mathbf{z}\left(\mathrm{t}\right)$ based on its initial value $\mathbf{z}\left(0\right)=0$ and referring to the state-space formulation. Given $\mathbf{z}\left(\mathrm{t}\right)$, the vectors ${\varvec{\uplambda}}\left(\mathrm{t}\right)$ and ${\varvec{\upeta}}\left(\mathrm{t}\right)$ are determined by a discrete backward-time scheme using the adjoint terms of Eqs. (37) and (41) while starting from the transversality conditions ${\varvec{\uplambda}}\left({\mathrm{t}}_{\mathrm{f}}\right)=0$ and ${\varvec{\upeta}}\left({\mathrm{t}}_{\mathrm{f}}\right)=0$. The proposed discrete-time scheme for their calculation is described herein.

For the fixed time-step size $\Delta t$, define the time index as ${t}_{i}=i\Delta t$ so that the final time index is $i=\frac{{t}_{f}}{\Delta t}$. For the final time step ${t}_{i}={t}_{f}$ of index $i=\frac{{t}_{f}}{\Delta t}$ define the following:

- $$\dot{{\varvec{\uplambda}}}\left({\mathrm{t}}_{\mathrm{f}}\right)=2\mathbf{Q}\mathbf{z}\left({\mathrm{t}}_{\mathrm{f}}\right)$$
- $${\varvec{\uplambda}}\left({\mathrm{t}}_{\mathrm{f}}\right)=0$$
- $$\dot{{\varvec{\upeta}}}\left({\mathrm{t}}_{\mathrm{f}}\right)=0$$
- $${\varvec{\upeta}}\left({\mathrm{t}}_{\mathrm{f}}\right)=0$$

Define ${\mathbf{A}\left(\mathbf{z}\right)}_{\mathrm{i}}=\mathbf{A}\left(\mathbf{z}\left({\mathrm{t}}_{\mathrm{i}}\right)\right)$. Then, in backward discrete-time $i=\frac{{t}_{f}}{\Delta t}-1,\dots ,0$ calculate ${\varvec{\uplambda}}\left({\mathrm{t}}_{\mathrm{i}}\right)$ and ${\varvec{\upeta}}\left({\mathrm{t}}_{\mathrm{i}}\right)$ by performing the following steps in the given order:

- $${\nabla }_{\mathbf{z}\left(\mathrm{t}\right)}{\mathbf{A}\left(\mathbf{z}\right)}_{\mathrm{i}}=\left({\mathbf{A}\left(\mathbf{z}\right)}_{\mathrm{i}+1}-{\mathbf{A}\left(\mathbf{z}\right)}_{\mathrm{i}-1}\right){\left(\mathbf{z}\left({\mathrm{t}}_{\mathrm{i}+1}\right)-\mathbf{z}\left({\mathrm{t}}_{\mathrm{i}-1}\right)\right)}^{-1}$$
- $${\widetilde{\mathbf{G}}}_{\mathrm{i}} \approx {\int }_{0}^{{\mathrm{t}}_{\mathrm{i}}}\mathbf{G}\left(\mathbf{w},\mathbf{z}\right)\mathrm{d\tau }={\int }_{0}^{{\mathrm{t}}_{\mathrm{i}}}\left[\begin{array}{cccc}0& 0& \mathbf{w}{\mathbf{k}}^{\mathrm{w}0}\left({\varvec{\updelta}},\dot{{\varvec{\updelta}}}\right)& 0\end{array}\right]\mathrm{d\tau }$$
- $${\widetilde{\mathbf{G}\mathbf{z}}}_{\mathrm{i}}\approx {\int }_{0}^{{\mathrm{t}}_{\mathrm{i}}}\mathbf{G}\left(\mathbf{w},\mathbf{z}\right)\mathbf{z}\left(\uptau \right)\mathrm{d\tau }={\int }_{0}^{{\mathrm{t}}_{\mathrm{i}}}\left[\begin{array}{cccc}0& 0& \mathbf{w}{\mathbf{k}}^{\mathrm{w}0}\left({\varvec{\updelta}},\dot{{\varvec{\updelta}}}\right)& 0\end{array}\right]\mathbf{z}\left(\uptau \right)\mathrm{d\tau }$$
- $${\mathbf{a}}_{1}=\mathbf{A}\left({\mathrm{t}}_{\mathrm{i}}\right)+{\nabla }_{\mathbf{z}\left(\mathrm{t}\right)}{\mathbf{A}\left(\mathbf{z}\right)}_{\mathrm{i}}\mathbf{z}\left({\mathrm{t}}_{\mathrm{i}}\right)-\mathbf{B}{\widetilde{\mathbf{G}}}_{\mathrm{i}}$$
- $${\mathbf{a}}_{2}=\frac{\mathrm{\Delta t}}{2}{\mathbf{a}}_{1}$$
- $${\mathbf{a}}_{3}={\mathbf{a}}_{2}-\mathbf{I}$$
- $$\dot{{\varvec{\uplambda}}}\left({\mathrm{t}}_{\mathrm{i}}\right)={{\mathbf{a}}_{3}}^{-1}\left({\mathbf{a}}_{1}{\varvec{\uplambda}}\left({\mathrm{t}}_{\mathrm{i}+1}\right)-{\mathbf{a}}_{2}\dot{{\varvec{\uplambda}}}\left({\mathrm{t}}_{\mathrm{i}+1}\right)\right)$$
- $${\varvec{\uplambda}}\left({\mathrm{t}}_{\mathrm{i}}\right)={\varvec{\uplambda}}\left({\mathrm{t}}_{\mathrm{i}+1}\right)-\frac{\mathrm{\Delta t}}{2}\left(\dot{{\varvec{\uplambda}}}\left({\mathrm{t}}_{\mathrm{i}+1}\right)+\dot{{\varvec{\uplambda}}}\left({\mathrm{t}}_{\mathrm{i}}\right)\right)$$
- $$\dot{{\varvec{\upeta}}}\left({\mathrm{t}}_{\mathrm{i}}\right)=-\mathrm{diag}\left\{{\mathbf{w}}^{-1}{\widetilde{\mathbf{G}\mathbf{z}}}_{\mathrm{i}}\right\}{\mathbf{B}}^{\mathrm{T}}{\varvec{\uplambda}}\left({\mathrm{t}}_{\mathrm{i}}\right)$$
- $${\varvec{\upeta}}\left({\mathrm{t}}_{\mathrm{i}}\right)={\varvec{\upeta}}\left({\mathrm{t}}_{\mathrm{i}+1}\right)-\frac{\mathrm{\Delta t}}{2}\left(\dot{{\varvec{\upeta}}}\left({\mathrm{t}}_{\mathrm{i}+1}\right)+\dot{{\varvec{\upeta}}}\left({\mathrm{t}}_{\mathrm{i}}\right)\right)$$

The proposed numeric evaluation uses standard numeric techniques—offering a stable paradigm. The calculation of ${\nabla }_{\mathbf{z}\left(\mathrm{t}\right)}{\mathbf{A}\left(\mathbf{z}\right)}_{\mathrm{i}}$ above uses the central difference scheme, and the integration of ${\varvec{\uplambda}}\left({\mathrm{t}}_{\mathrm{i}}\right)$ and ${\varvec{\upeta}}\left({\mathrm{t}}_{\mathrm{i}}\right)$ is based on the extended mean value theorem. Also, regarding the numeric terms of steps (b) and (c), which refer to the numeric integration technique, it is recommended to solve these terms using Simpson's rule due to its accuracy and idealized scheme.

## SMARD design procedure

Having defined the gradient ${\widetilde{\mathrm{J}}}_{\widehat{\mathbf{w}}}\left(\mathbf{w}\right)$, the optimal wires quantity is attained by minimizing the objective function $\mathrm{J}\left(\mathbf{w}\right)$ iteratively. The design algorithm for SMARDs is composed of nine steps and is provided herein.

- Define the inelastic shear-type frame's structural properties (e.g., ${m}_{n}$, ${k}_{n}^{el}$, ${f}_{n}^{yld}$, ${a}_{n}$) and SMARD properties (i.e., ${E}_{n}^{SMA}$, ${\sigma }_{n}^{SMA,yld}$, ${f}^{T}$, ${\alpha }_{n}^{SMA}$)
- Define the ground acceleration sequence ${a}_{g}\left(t\right)$
- Decide the algorithm parameters: the optimal weighting matrix $\mathbf{Q}$, and the minimum/maximum number of SMA wires assigned to each story. That is:
  $$\begin{array}{ccc}{\mathbf{w}}^{\mathrm{min}}=\left[\begin{array}{c}{\mathrm{w}}_{1}^{\mathrm{min}}\\ \vdots \\ {\mathrm{w}}_{\mathrm{N}}^{\mathrm{min}}\end{array}\right]& \mathrm{and}& {\mathbf{w}}^{\mathrm{max}}=\left[\begin{array}{c}{\mathrm{w}}_{1}^{\mathrm{max}}\\ \vdots \\ {\mathrm{w}}_{\mathrm{N}}^{\mathrm{max}}\end{array}\right]\end{array}$$
- Set the iteration index to k = 0 and choose the initial number of SMA wires assigned to the SMARDs in each story:
  $${\mathbf{w}}^{0}=\left[\begin{array}{c}{\mathrm{w}}_{1}^{0}\\ \vdots \\ {\mathrm{w}}_{\mathrm{N}}^{0}\end{array}\right]$$
- Calculate the state vector ${\mathbf{z}}^{\mathrm{k}}\left(\mathrm{t}\right)$ by solving the initial value problem:
  $$\begin{array}{l}{\dot{\mathbf{z}}}^{\mathrm{k}}\left(\mathrm{t}\right)=\mathbf{A}\left({\mathbf{z}}^{\mathrm{k}}\left(\mathrm{t}\right)\right){\mathbf{z}}^{\mathrm{k}}\left(\mathrm{t}\right)+\mathbf{B}\left(\mathrm{t}\right)\left(-\mathbf{m}{\mathbf{e}}_{1}{\mathrm{a}}_{\mathrm{g}}\left(\mathrm{t}\right)+\mathbf{u}\left(\mathbf{w},\mathbf{z}\left(\mathrm{t}\right)\right)\right)\\ {\mathbf{z}}^{\mathrm{k}}\left(0\right)=0\end{array}$$
- Calculate for ${{\varvec{\uplambda}}}^{\mathrm{k}}\left(\mathrm{t}\right)$ and ${{\varvec{\upeta}}}^{\mathrm{k}}\left(\mathrm{t}\right)$ using the backward-discrete-time scheme presented in “Numeric evaluation of λ(t) and η(t)” and come up with ${{\varvec{\upeta}}}^{\mathrm{k}}\left(0\right)$
- If k = 0, proceed to the next step.
  If k > 0 and if:
  $${\upeta }_{\mathrm{n}}^{\mathrm{k}}\left(0\right){\upeta }_{\mathrm{n}}^{\mathrm{k}-1}\left(0\right)<0$$
  then $${\mathrm{w}}_{\mathrm{n}}^{\mathrm{k}}={\widehat{\mathrm{w}}}_{\mathrm{n}}^{\mathrm{k}}$$
- If $${\mathrm{w}}_{\mathrm{n}}^{\mathrm{k}}\ne {\widehat{\mathrm{w}}}_{\mathrm{n}}^{\mathrm{k}}$$, then:
  $${\mathrm{w}}_{\mathrm{n}}^{\mathrm{k}+1}=\left\{\begin{array}{cccc}{\mathrm{w}}_{\mathrm{n}}^{\mathrm{k}}-\mathrm{sign}\left({\upeta }_{\mathrm{n}}^{\mathrm{k}}\left(0\right)\right)& ,& {\mathrm{w}}_{\mathrm{n}}^{\mathrm{k}}>{\mathrm{w}}_{\mathrm{n}}^{\mathrm{min}} & {\mathrm{w}}_{\mathrm{n}}^{\mathrm{k}}<{\mathrm{w}}_{\mathrm{n}}^{\mathrm{max}}\\ {\mathrm{w}}_{\mathrm{n}}^{\mathrm{k}}-1& ,& {\mathrm{w}}_{\mathrm{n}}^{\mathrm{k}}={\mathrm{w}}_{\mathrm{n}}^{\mathrm{min}} & \mathrm{sign}\left({\upeta }_{\mathrm{n}}^{\mathrm{k}}\left(0\right)\right)>0\\ {\mathrm{w}}_{\mathrm{n}}^{\mathrm{k}}+1& ,& {\mathrm{w}}_{\mathrm{n}}^{\mathrm{k}}={\mathrm{w}}_{\mathrm{n}}^{\mathrm{max}} & \mathrm{sign}\left({\upeta }_{\mathrm{n}}^{\mathrm{k}}\left(0\right)\right)<0\end{array}\right.$$
- If ${\mathbf{w}}^{\mathrm{k}+1}\ne {\mathbf{w}}^{\mathrm{k}}$ go back to step (e). Else, finish.

## Numerical example

**Figure 4:**
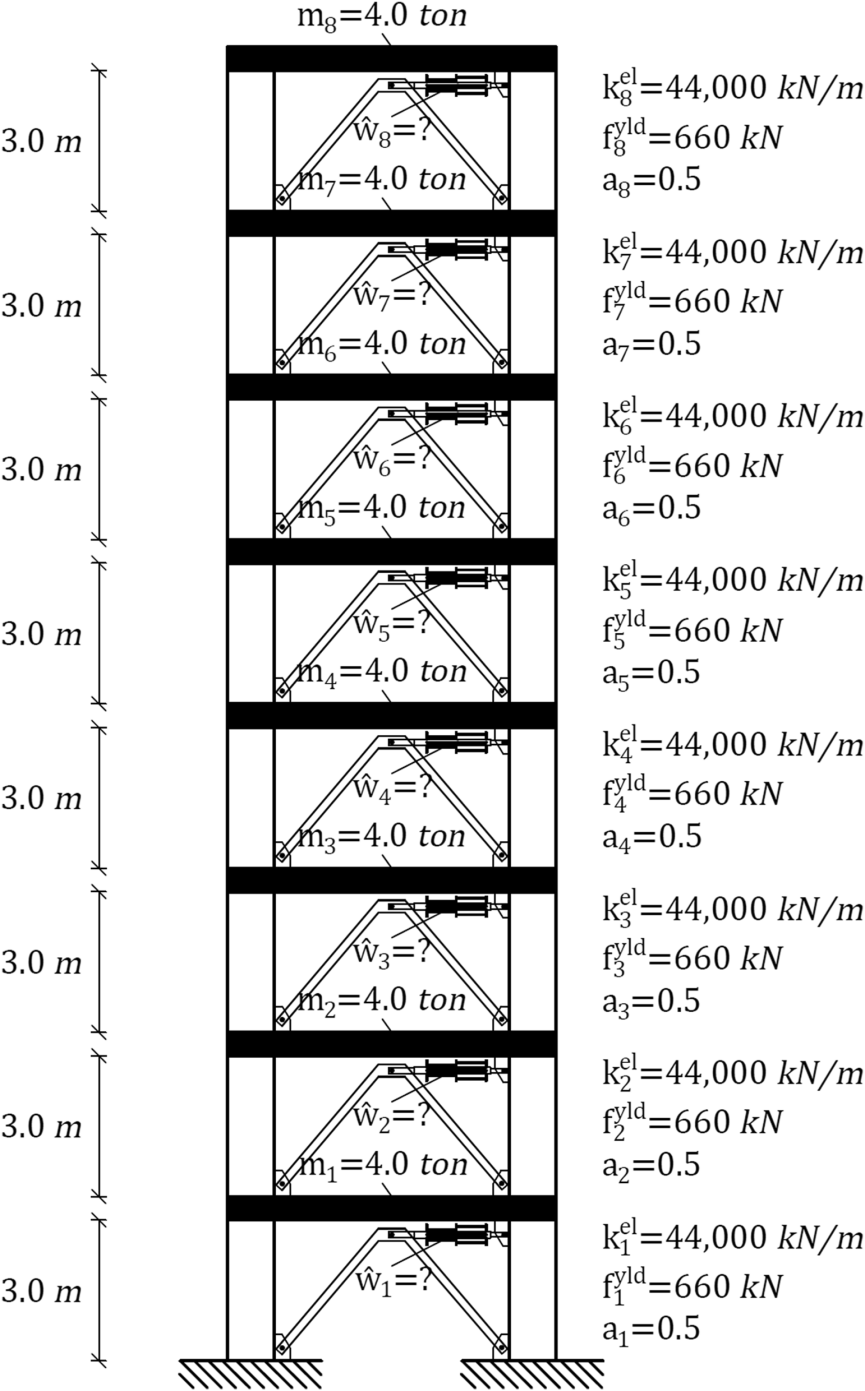
Eight-story inelastic shear-type system.

The numerical example examines the SMARD design algorithm by analyzing and retrofitting an eight-story shear-type frame system. Figure 4 depicts the system elevation scheme and specifies the stories' columns length, ceiling mass quantity ${m}_{n}$, lateral stiffness ${k}_{n}^{el}$, resisting shear force at first yield ${f}_{n}^{yld}$, and the ratios between the plastic and elastic stiffness ${a}_{n}$. The columns' hysteretic model assumes symmetric yielding, no stiffness degradation, no strength degradation, and pinching effect. Also, an inherent damping ratio of 5% is considered. It is noted that the parametric notations presented herein are defined and explained in “Hysteretic SMA model”.

The SMA properties are taken from Wilde et al.^27^. Accordingly, the cyclic SMA model parameters are ${\mathrm{f}}^{\mathrm{T}}=0.08$, ${\upgamma }_{1}=1.0$, and ${\upgamma }_{2}=0.001$. The SMA material and geometrical properties are:

$$\begin{array}{ccc}\left.\begin{array}{c}{\mathrm{L}}_{\mathrm{n}}^{\mathrm{SMA}}=1.0 m\\ {\mathrm{A}}_{\mathrm{n}}^{\mathrm{SMA}}=0.0254 {cm}^{2}\\ {\mathrm{E}}_{\mathrm{n}}^{\mathrm{SMA}}={9.8325\cdot 10}^{7}\frac{kN}{{m}^{2}}\\ {\mathrm{E}}_{\mathrm{n}}^{\mathrm{m},\mathrm{SMA}}={7.3744\cdot 10}^{7}\frac{kN}{{m}^{2}}\\ {\mathrm{\alpha }}_{\mathrm{n}}^{\mathrm{SMA}}=0.0197\\ {\mathrm{a}}^{\mathrm{SMA}}=900\\ {\upsigma }_{\mathrm{n}}^{\mathrm{SMA},\mathrm{yld}}=14.5\cdot {10}^{4}\frac{kN}{{m}^{2}}\\ {\upepsilon }_{\mathrm{n}}^{1,\mathrm{SMA}}=0.05\\ {\upepsilon }_{\mathrm{n}}^{\mathrm{m},\mathrm{SMA}}=0.08\end{array}\right\}& \forall & \mathrm{n}=1,\dots ,\mathrm{N}\end{array}$$

It might be worth noting that indoor experiments determined the SMA material parameters. Accordingly, the utilization of SMARDs for retrofitting frame systems is also indoors. Under the assumption the room temperature does not change drastically, these experiment-based parameters are suitable for this paper's case.

In this example, the design algorithm addresses a sinusoidal ground acceleration sequence of displacement resonant frequency:

$$\upomega ={\upomega }_{1}\sqrt{1-2{\left({\upzeta }_{1}\right)}^{2}}=19.35\sqrt{1-2{\left(0.05\right)}^{2}}=19.3 \mathrm{rad}/\mathrm{sec}$$

where ${\omega }_{1}$ is the lowest modal frequency of the shear-type system, and ${\zeta }_{1}=0.05$ is the modal system's damping ratio. The peak ground acceleration value is 1.0* g,* and the loading time is defined as 13.35* s* to have an Arias Intensity level of 10* m/s*. Accordingly, the ground acceleration function is:

$$\begin{array}{ccc}{\mathrm{a}}_{\mathrm{g}}\left(\mathrm{t}\right)=1.0\mathrm{sin}\left(19.3\mathrm{t}\right)& \leftrightarrow & \mathrm{t}\in \left[\begin{array}{cc}0& 13.35\end{array}\right]\end{array}$$

The minimum and the maximum SMA wire limitations are prescribed as 0 and 150 for all stories. Therefore, ${\mathbf{w}}^{\mathrm{min}}$ and ${\mathbf{w}}^{\mathrm{max}}$ are defined as:

$$\begin{array}{ccc}{\mathbf{w}}^{\mathrm{min}}=0& \mathrm{and}& {\mathbf{w}}^{\mathrm{max}}=150\mathbf{I}\end{array}$$

The numerical study focuses and expands on the effect of the weighting matrix $\mathbf{Q}$ on the solution of $\widehat{\mathbf{w}}$ and studies three different configurations. The matrix $\mathbf{Q}$ is analyzed using the *N* × *N* sub-matrices ${\mathbf{Q}}_{1}$, ${\mathbf{Q}}_{2}$, ${\mathbf{Q}}_{3}$, ${\mathbf{Q}}_{4}$ that relate to ${\varvec{\updelta}}\left(\mathrm{t}\right)$, ${\int }_{0}^{\mathrm{t}}{\mathbf{f}}^{\mathrm{H}}\left(\uptau \right)\mathrm{d\tau }$, $\dot{{\varvec{\updelta}}}\left(\mathrm{t}\right)$, and ${\mathbf{f}}^{\mathrm{H}}\left(\mathrm{t}\right)$, respectively, so that:

$$\mathbf{Q}=\left[\begin{array}{cccc}{\mathbf{Q}}_{1}& & & \\ & {\mathbf{Q}}_{2}& & \\ & & {\mathbf{Q}}_{3}& \\ & & & {\mathbf{Q}}_{4}\end{array}\right]$$

The developed design algorithm's objective is to minimize the functional ${\int }_{0}^{{\mathrm{t}}_{\mathrm{f}}}{\mathbf{z}\left(\mathrm{t}\right)}^{\mathrm{T}}\mathbf{Q}\mathbf{z}\left(\mathrm{t}\right)\mathrm{dt}$. In examining the effectiveness of the weighting sub-matrices, we quantify and compare between the application of time integral to the squared components of $\mathbf{z}\left(\mathrm{t}\right)$:

$${\mathbf{i}\mathbf{n}\mathbf{t}}^{\mathrm{I}}={\int }_{0}^{{\mathrm{t}}_{\mathrm{f}}}{\left({\varvec{\updelta}}\left(\mathrm{t}\right)\right)}^{2}\mathrm{dt}$$

$${\mathbf{i}\mathbf{n}\mathbf{t}}^{\mathrm{II}}={\int }_{0}^{{\mathrm{t}}_{\mathrm{f}}}{\left({\int }_{0}^{\mathrm{t}}{\mathbf{f}}^{\mathrm{H}}\left(\uptau \right)\mathrm{d\tau }\right)}^{2}\mathrm{dt}$$

$${\mathbf{i}\mathbf{n}\mathbf{t}}^{\mathrm{III}}={\int }_{0}^{{\mathrm{t}}_{\mathrm{f}}}{\left(\dot{{\varvec{\updelta}}}\left(\mathrm{t}\right)\right)}^{2}\mathrm{dt}$$

$${\mathbf{i}\mathbf{n}\mathbf{t}}^{\mathrm{IV}}={\int }_{0}^{{\mathrm{t}}_{\mathrm{f}}}{\left({\mathbf{f}}^{\mathrm{H}}\left(\mathrm{t}\right)\right)}^{2}\mathrm{dt}$$

The resultant integration vectors are referred to herein as the system performance vectors. ∫

In the first optimization case, the sub-matrices ${\mathbf{Q}}_{1}$, ${\mathbf{Q}}_{2}$, ${\mathbf{Q}}_{3}$, ${\mathbf{Q}}_{4}$ are all defined as $\mathbf{I}$, by default, to comprehend the relative sizes between the consequent integration vectors ${\mathbf{i}\mathbf{n}\mathbf{t}}^{\mathrm{I}}$, ${\mathbf{i}\mathbf{n}\mathbf{t}}^{\mathrm{II}}$, ${\mathbf{i}\mathbf{n}\mathbf{t}}^{\mathrm{III}}$, and ${\mathbf{i}\mathbf{n}\mathbf{t}}^{\mathrm{IV}}$. The initial number of SMA wires assigned to all stories is 75—the average between the minimum and maximum quantities:

$${\mathbf{w}}^{0}=0.5\left({\mathbf{w}}^{\mathrm{min}}+{\mathbf{w}}^{\mathrm{max}}\right)=\left[\begin{array}{ccc}75& & \\ & \ddots & \\ & & 75\end{array}\right]$$

**Figure 5:**
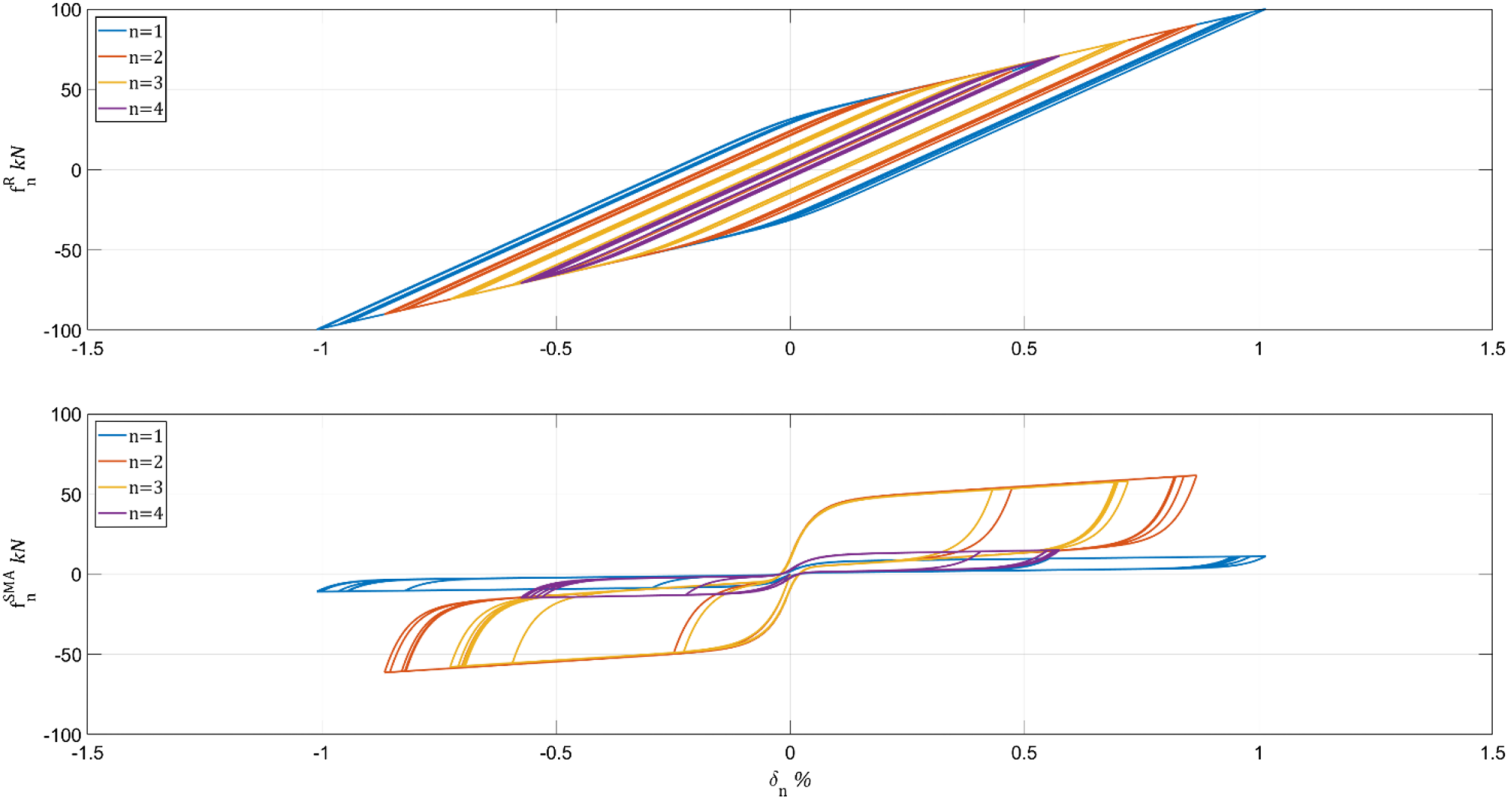
Hysteretic behavior of final SMA allocation with ${\mathbf{Q}}_{1}=\mathbf{I}$, ${\mathbf{Q}}_{2}=\mathbf{I}$, ${\mathbf{Q}}_{3}=\mathbf{I}$, ${\mathbf{Q}}_{4}=\mathbf{I}$

Table 1 shows the system performance results for ${\mathbf{w}}^{0}$ (without optimization). Applying the design algorithm's iterative steps (e)–(i) to the initial retrofit, after 69 iterations, results in the optimal number of SMA wires and system performance shown in Table 2. A comparison between Tables 1 and 2 shows that the design algorithm has reduced the total number of SMA wires from 600 to 548 while slightly deterring the system's performance. The hysteretic behavior of the Table 2 solution is depicted in Fig. 5 for stories 1–4.

**Table 1:** No optimization.

n	${\mathrm{w}}_{\mathrm{n}}$	${10}^{4} {{\mathrm{int}}^{\mathrm{I}}}_{\mathrm{n}},$ m^2^s	${10}^{-2} {{\mathrm{int}}^{\mathrm{II}}}_{\mathrm{n}},$ kN^2^s^3^	${10}^{1} {{\mathrm{int}}^{\mathrm{III}}}_{\mathrm{n}},$ m^2^/s	${10}^{-5} {{\mathrm{int}}^{\mathrm{IV}}}_{\mathrm{n}},$ kN^2^s
8	75	0.90	1.08	0.81	0.43
7	75	3.80	4.71	2.89	1.83
6	75	8.09	10.32	4.92	3.86
5	75	13.01	17.87	6.29	6.05
4	75	18.37	23.45	7.36	7.94
3	75	27.27	26.28	10.35	8.85
2	75	35.78	27.54	14.23	9.66
1	75	40.29	30.11	16.61	10.20
Sum	600	147.50	141.37	63.54	48.81

**Table 2:** Identity weighting sub-matrices (default choice): ${\mathbf{Q}}_{1}=\mathbf{I}$, ${\mathbf{Q}}_{2}=\mathbf{I}$, ${\mathbf{Q}}_{3}=\mathbf{I}$, ${\mathbf{Q}}_{4}=\mathbf{I}$

n	${\widehat{\mathrm{w}}}_{\mathrm{n}}$	${10}^{4} {{\mathrm{int}}^{\mathrm{I}}}_{\mathrm{n}},$ m^2^s	${10}^{-2} {{\mathrm{int}}^{\mathrm{II}}}_{\mathrm{n}},$ kN^2^s^3^	${10}^{1} {{\mathrm{int}}^{\mathrm{III}}}_{\mathrm{n}},$ m^2^/s	${10}^{-5} {{\mathrm{int}}^{\mathrm{IV}}}_{\mathrm{n}},$ kN^2^s
8	26	1.11	1.31	1.17	0.53
7	40	4.14	5.17	3.38	1.99
6	66	8.29	10.50	5.12	3.95
5	106	12.56	16.34	6.08	5.91
4	34	19.92	24.10	8.08	8.15
3	126	25.30	25.75	9.85	8.73
2	128	32.76	26.75	13.54	9.57
1	22	44.58	31.29	17.86	10.33
Sum	548	148.65	141.21	65.08	49.15

The system performance vectors greatly vary in size. For the subsequent examination, The weighting sub-matrices are changed into ${\mathbf{Q}}_{1}=1{0}^{9}\mathbf{I}$, ${\mathbf{Q}}_{2}=1{0}^{3}\mathbf{I}$, ${\mathbf{Q}}_{3}=1{0}^{6}\mathbf{I}$, and ${\mathbf{Q}}_{4}=\mathbf{I}$ to equalize the functions that construct the objective function. That is, considering:

$$\mathrm{J}={\int }_{0}^{{\mathrm{t}}_{\mathrm{f}}}{{\varvec{\updelta}}\left(\mathrm{t}\right)}^{\mathrm{T}}{\mathbf{Q}}_{1}{\varvec{\updelta}}\left(\mathrm{t}\right)\mathrm{dt}+{\int }_{0}^{{\mathrm{t}}_{\mathrm{f}}}{\left({\int }_{0}^{\mathrm{t}}{\mathbf{f}}^{\mathrm{H}}\left(\uptau \right)\mathrm{d\tau }\right)}^{\mathrm{T}}{\mathbf{Q}}_{2}\left({\int }_{0}^{\mathrm{t}}{\mathbf{f}}^{\mathrm{H}}\left(\uptau \right)\mathrm{d\tau }\right)\mathrm{dt}+\dots {\int }_{0}^{{\mathrm{t}}_{\mathrm{f}}}{\dot{{\varvec{\updelta}}}\left(\mathrm{t}\right)}^{\mathrm{T}}{\mathbf{Q}}_{3}\dot{{\varvec{\updelta}}}\left(\mathrm{t}\right)\mathrm{dt}+{\int }_{0}^{{\mathrm{t}}_{\mathrm{f}}}{\left({\mathbf{f}}^{\mathrm{H}}\left(\mathrm{t}\right)\right)}^{\mathrm{T}}{\mathbf{Q}}_{4}\left({\mathbf{f}}^{\mathrm{H}}\left(\mathrm{t}\right)\right)\mathrm{dt}$$

With the current weighting sub-matrices, we have:

$${\int }_{0}^{{\mathrm{t}}_{\mathrm{f}}}{{\varvec{\updelta}}\left(\mathrm{t}\right)}^{\mathrm{T}}{\mathbf{Q}}_{1}{\varvec{\updelta}}\left(\mathrm{t}\right)\mathrm{dt}\sim {\int }_{0}^{{\mathrm{t}}_{\mathrm{f}}}{\left({\int }_{0}^{\mathrm{t}}{\mathbf{f}}^{\mathrm{H}}\left(\uptau \right)\mathrm{d\tau }\right)}^{\mathrm{T}}{\mathbf{Q}}_{2}\left({\int }_{0}^{\mathrm{t}}{\mathbf{f}}^{\mathrm{H}}\left(\uptau \right)\mathrm{d\tau }\right)\mathrm{dt}\sim {\int }_{0}^{{\mathrm{t}}_{\mathrm{f}}}{\dot{{\varvec{\updelta}}}\left(\mathrm{t}\right)}^{\mathrm{T}}{\mathbf{Q}}_{3}\dot{{\varvec{\updelta}}}\left(\mathrm{t}\right)\mathrm{dt}\sim {\int }_{0}^{{\mathrm{t}}_{\mathrm{f}}}{\left({\mathbf{f}}^{\mathrm{H}}\left(\mathrm{t}\right)\right)}^{\mathrm{T}}{\mathbf{Q}}_{4}\left({\mathbf{f}}^{\mathrm{H}}\left(\mathrm{t}\right)\right)\mathrm{dt}$$

**Figure 6:**
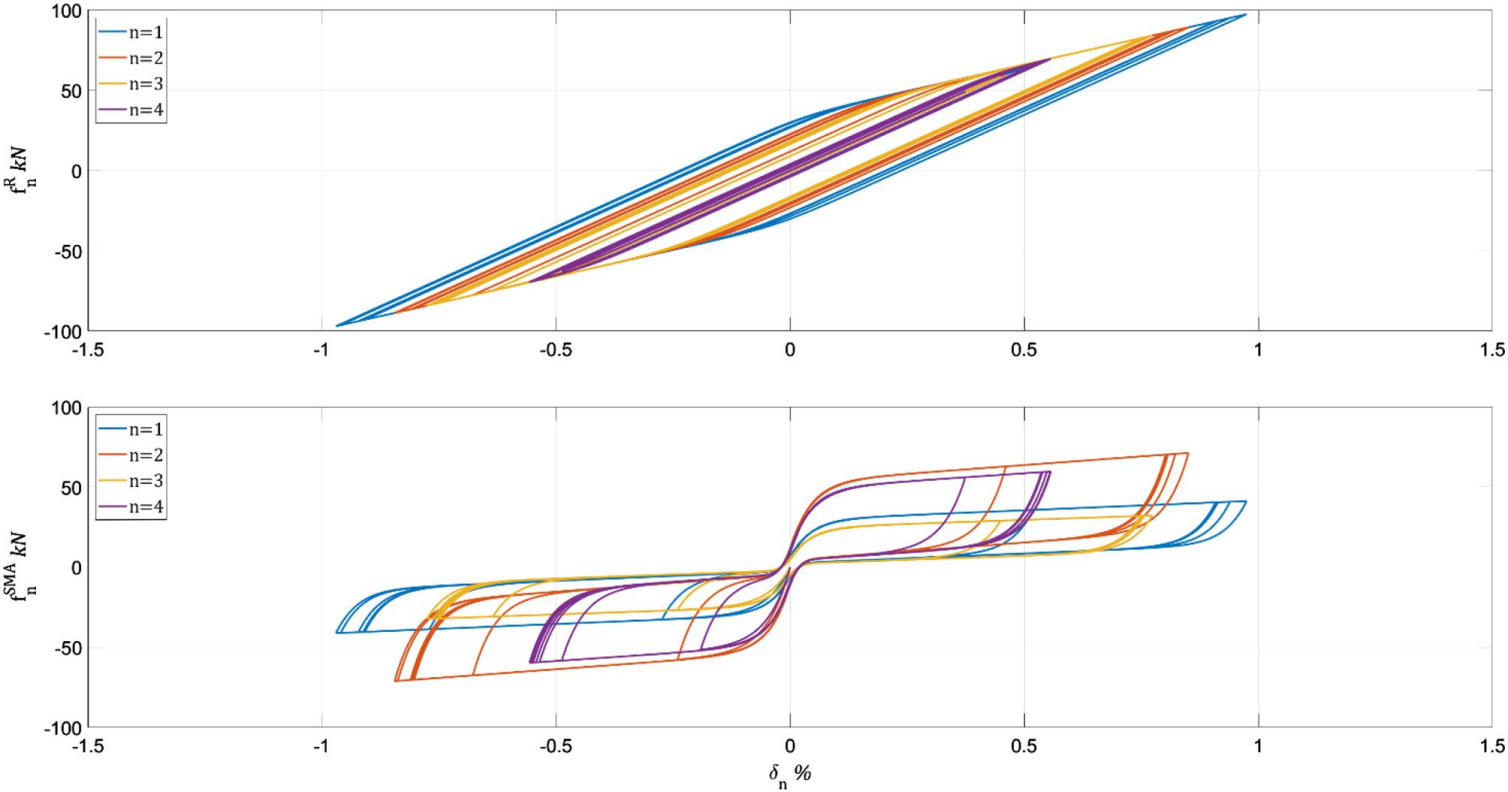
Hysteretic behavior of final SMA allocation with ${\mathbf{Q}}_{1}=1{0}^{9}\mathbf{I}$, ${\mathbf{Q}}_{2}=1{0}^{3}\mathbf{I}$, ${\mathbf{Q}}_{3}=1{0}^{6}\mathbf{I}$, ${\mathbf{Q}}_{4}=\mathbf{I}$

The algorithm needed 98 iterations to converge into the system performance shown in Table 3. The algorithm attains a more efficient design for SMA wires, decreasing the wire's total from 600 to 534 while slightly increasing the system performance. The hysteretic behavior of the Table 3 solution is depicted in Fig. 6 for stories 1–4.

**Table 3:** Equalized weighting sub-matrices: ${\mathbf{Q}}_{1}=1{0}^{9}\mathbf{I}$, ${\mathbf{Q}}_{2}=1{0}^{3}\mathbf{I}$, ${\mathbf{Q}}_{3}=1{0}^{6}\mathbf{I}$, ${\mathbf{Q}}_{4}=\mathbf{I}$

n	${\widehat{\mathrm{w}}}_{\mathrm{n}}$	${10}^{4} {{\mathrm{int}}^{\mathrm{I}}}_{\mathrm{n}},$ m^2^s	${10}^{-2} {{\mathrm{int}}^{\mathrm{II}}}_{\mathrm{n}},$ kN^2^s^3^	${10}^{1} {{\mathrm{int}}^{\mathrm{III}}}_{\mathrm{n}},$ m^2^/s	${10}^{-5} {{\mathrm{int}}^{\mathrm{IV}}}_{\mathrm{n}},$ kN^2^s
8	0	1.25	1.51	1.32	0.60
7	27	4.37	5.38	3.57	2.10
6	0	9.24	12.05	5.66	4.36
5	69	13.63	17.75	6.41	6.26
4	137	17.87	21.98	7.21	7.74
3	69	29.70	26.97	11.40	9.03
2	149	32.73	27.64	13.26	9.71
1	83	41.53	29.78	16.82	10.41
Sum	534	150.32	143.06	65.65	50.19

Lastly, in the third case, we look at a point where the algorithm shows improvement in all system performance vectors. Accordingly, weighting sub-matrices are defined to prioritize the minimization of $\dot{{\varvec{\updelta}}}\left(\mathrm{t}\right)$—a performance that relates to the cumulative expressions of all other state vector components:

$${\varvec{\updelta}}\left(\mathrm{t}\right)={\int }_{0}^{\mathrm{t}}\dot{{\varvec{\updelta}}}\left(\uptau \right)\mathrm{d\tau }$$

$${\mathbf{f}}^{\mathrm{H}}\left(\mathrm{t}\right)={\int }_{0}^{\mathrm{t}}{\dot{\mathbf{f}}}^{\mathrm{H}}\left(\uptau \right)\mathrm{d\tau }={\int }_{0}^{\mathrm{t}}{\mathbf{k}}^{\mathrm{H}}\left({\mathbf{f}}^{\mathrm{H}},\dot{{\varvec{\updelta}}}\right) \dot{{\varvec{\updelta}}}\left(\uptau \right)\mathrm{d\tau }$$

$${\int }_{0}^{\mathrm{t}}{\mathbf{f}}^{\mathrm{H}}\left(\uptau \right)\mathrm{d\tau }={\int }_{0}^{\mathrm{t}}{\int }_{0}^{\uptau }{\mathbf{k}}^{\mathrm{H}}\left({\mathbf{f}}^{\mathrm{H}},\dot{{\varvec{\updelta}}}\right) \dot{{\varvec{\updelta}}}\left(\upnu \right)\mathrm{d\nu d\tau }$$

**Figure 7:**
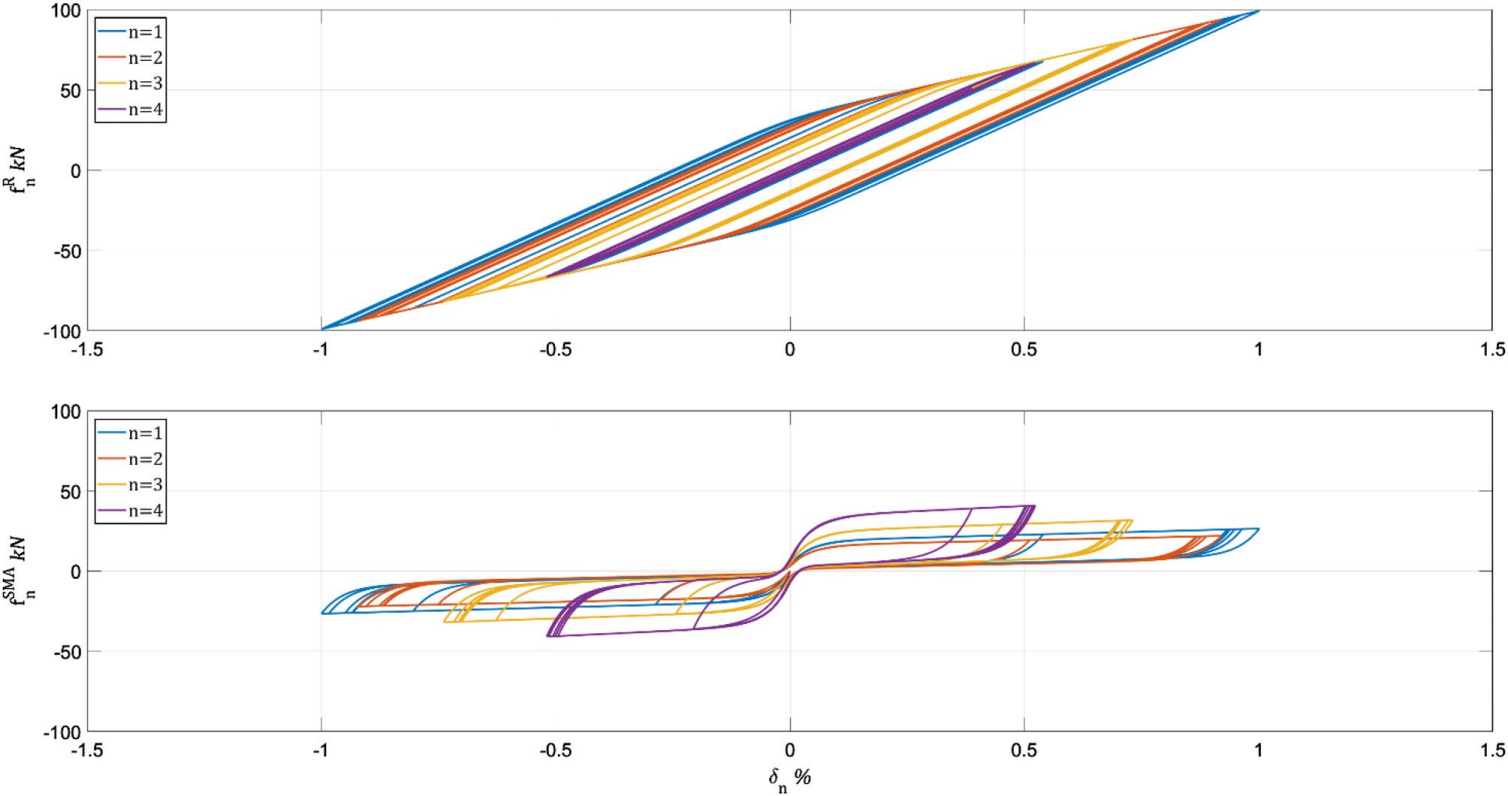
Hysteretic behavior of final SMA allocation with ${\mathbf{Q}}_{1}=\mathbf{I}$, ${\mathbf{Q}}_{2}=\mathbf{I}$, ${\mathbf{Q}}_{3}=1{0}^{12}\mathbf{I}$, ${\mathbf{Q}}_{4}=\mathbf{I}$

The prioritization is brought about by defining ${\mathbf{Q}}_{1}=\mathbf{I}$, ${\mathbf{Q}}_{2}=\mathbf{I}$, ${\mathbf{Q}}_{3}=1{0}^{12}\mathbf{I}$, and ${\mathbf{Q}}_{4}=\mathbf{I}$. After 30 iterations, the algorithm met the design solution shown in Table 4. The design algorithm minimizes the sum of system performance vector components by increasing the SMA wires from 600 to 620. Figure 7 depicts the hysteretic behavior of the Table 3 solution for stories 1–4.

**Table 4:** Results when $\dot{{\varvec{\updelta}}}\left(\mathbf{t}\right)$ minimization is prioritized: ${\mathbf{Q}}_{1}=\mathbf{I}$, ${\mathbf{Q}}_{2}=\mathbf{I}$, ${\mathbf{Q}}_{3}=1{0}^{12}\mathbf{I}$, ${\mathbf{Q}}_{4}=\mathbf{I}$

n	${\widehat{\mathrm{w}}}_{\mathrm{n}}$	${10}^{4} {{\mathrm{int}}^{\mathrm{I}}}_{\mathrm{n}},$ m^2^s	${10}^{-2} {{\mathrm{int}}^{\mathrm{II}}}_{\mathrm{n}},$ kN^2^s^3^	${10}^{1} {{\mathrm{int}}^{\mathrm{III}}}_{\mathrm{n}},$ m^2^/s	${10}^{-5} {{\mathrm{int}}^{\mathrm{IV}}}_{\mathrm{n}},$ kN^2^s
8	97	0.81	0.96	0.72	0.39
7	101	3.54	4.34	2.72	1.70
6	97	7.70	9.87	4.82	3.69
5	63	12.92	16.97	6.46	6.01
4	95	17.17	22.79	7.01	7.74
3	69	26.47	26.05	10.09	8.78
2	45	36.76	27.83	14.64	9.60
1	53	41.01	29.96	17.07	10.14
Sum	620	146.38	138.77	63.53	48.05

The results shown in Table 4 are compared with the optimal SMARD allocation of Mulay and Shmerling^21^. Their method takes s a predefined SMARD configuration and finds the optimal story (using their extended transformation matrix). That process repeats until all SMARDs are placed. Considering the SMARD configurations of Table 4, the method of^20^ results in the design shown in Table 5 and favorites placing the SMARDs in the lower stories. In terms of minimal time-integration trajectories, the results in Table 5 are superior in all meanings.

**Table 5:** Optimal design by Mulay and Shmerling^21^.

n	${\widehat{\mathrm{w}}}_{\mathrm{n}}$	${10}^{4} {{\mathrm{int}}^{\mathrm{I}}}_{\mathrm{n}}$, m^2^s	${10}^{-2} {{\mathrm{int}}^{\mathrm{II}}}_{\mathrm{n}}$, kN^2^s^3^	${10}^{1} {{\mathrm{int}}^{\mathrm{III}}}_{\mathrm{n}}$, m^2^/s	${10}^{-5} {{\mathrm{int}}^{\mathrm{IV}}}_{\mathrm{n}}$, kN^2^s
8	0	1.28	1.59	1.21	0.61
7	0	4.72	6.05	3.43	2.27
6	0	9.63	13.34	5.35	4.54
5	0	15.95	20.34	7.16	6.84
4	101	20.93	23.47	8.14	8.06
3	194	25.49	26.05	9.78	8.88
2	132	36.84	28.28	14.36	9.91
1	193	36.69	28.91	14.95	10.28
Sum	620	151.52	148.03	64.39	51.38

## Conclusions

A new design methodology for using SMARDs to regulate seismic vibrations is presented. The modified constitutive law for SMA material expresses the SMARDs' tangent stiffness and extracts the number of SMA wires as the design variables. The design approach is an iterative procedure that solves an optimization problem inelastic systems' control gain. The objective function is subject to the state-space representation and design limitation on the minimum/maximum SMA wires. In formulating the state-space equation of the inelastic system, a new form of the state vector is proposed, which consists of the interstory drifts vector, the time integral of the hysteretic portion of the resisting shear force vector, the drift velocities vector, and the hysteretic part of the resisting shear force vector. The proposed state vector corresponds to an idealized state matrix that accounts for the system's inelastic behavior.

Pontryagin's Hamiltonian is employed to formulate the necessary conditions for optimizing the objective function and defining the objective function's steepest gradient. Following the gradient descent convergences into a local minimum, which depends on the initial optimized gains. Numeric evaluation schemes are proposed for calculating the Lagrange multipliers and the Hamiltonian's derivatives while adhering to all the optimality conditions. Idealized numeric techniques yield a stable and efficient calculation process, such as the central difference formula and the extended mean value theorem. The SMARD design algorithm utilizes the steepest descent approach. The objective function gradient is developed, and the optimal SMA wires solution is attained using a line-search strategy.

The numerical example retrofits an eight-story shear-type frame system, studies the SMARD design algorithm using three different weighting matrix configurations, and looks at the consequent system performance and total SMA wires. The employed dynamic load is a ground acceleration sequence of displacement resonant frequency and large PGA and Arias intensity levels. According to the three weighting matrix cases, the best result is attained in system performance when the interstory drift velocities are preferred. However, in terms of total SMA wires (i.e., retrofit resources), the best solution is achieved when the weighting matrix components are equalized in magnitudes.

## Data Availability

The datasets generated during and/or analysed during the current study are available from the corresponding author on reasonable request.

## References

[1] Khodaverdian A, Ghorbani-Tanha AK, Rahimian M (2012). An innovative base isolation system with Ni-Ti alloy and its application in seismic vibration control of Izadkhast Bridge. J. Intell. Mater. Syst. Struct..

[2] Kumbhar SB, Chavan SP, Gawade SS (2018). Adaptive tuned vibration absorber based on magnetorheological elastomer-shape memory alloy composite. Mech. Syst. Signal Process..

[3] Yang X, Hong J, Ma YH, Zhang DY (2012). Active vibration control of a double-decker cantilever beam using shape memory alloy. Appl. Mech. Mater..

[4] Ozbulut OE, Hurlebaus S (2011). Energy-balance assessment of shape memory alloy-based seismic isolation devices. Smart Struct. Syst..

[5] Gur S, Mishra SK, Bhowmick S, Chakraborty S (2014). Compliant liquid column damper modified by shape memory alloy device for seismic vibration control. Smart Mater. Struct..

[6] Shinozuka M, Chaudhuri SR, Mishra SK (2015). Shape-memory-alloy supplemented lead rubber bearing (SMA-LRB) for seismic isolation. Probab. Eng. Mech..

[7] Janke L, Czaderski C, Motavalli M, Ruth J (2005). Applications of shape memory alloys in civil engineering structures—Overview, limits and new ideas. Mater. Struct. Constr..

[8] Dong J, Cai CS, Okeil AM (2011). Overview of potential and existing applications of shape memory alloys in bridges. J. Bridg. Eng..

[9] Li, S., Hedayati Dezfuli, F., Wang, J. & Alam, M. S. Performance-based seismic loss assessment of isolated simply-supported highway bridges retrofitted with different shape memory alloy cable restrainers in a life-cycle context. *J. Intell. Mater. Syst. Struct.* **31**, 1053–1075 (2020).

[10] Rele R, Balmukund R, Bhattacharya S, Cui L, Mitoulis SA (2021). Application of controlled-rocking isolation with shape memory alloys for an overpass bridge. Soil Dyn. Earthq. Eng..

[11] Cao S, Yi J (2021). Shape memory alloy-spring damper for seismic control and its application to bridge with laminated rubber bearings. Adv. Struct. Eng..

[12] Vůjtěch J, Ryjáček P, Campos Matos J, Ghafoori E (2021). Iron-Based shape memory alloy for strengthening of 113-Year bridge. Eng. Struct..

[13] Han YL, Li QS, Li AQ, Leung AYT, Lin PH (2003). Structural vibration control by shape memory alloy damper. Earthq. Eng. Struct. Dyn..

[14] Mondal PD, Ghosh AD, Chakraborty S (2017). Control of underground blast induced building vibration by shape-memory-alloy rubber bearing (SMARB). Struct. Control Heal. Monit..

[15] Bubner N, Sokołowski J, Sprekels J (1998). Optimal boundary control problems for shape memory alloys under state constraints for stress and temperature. Numer. Funct. Anal. Optim..

[16] Piccirillo V, Balthazar JM, Pontes BR, Felix JLP (2009). Chaos control of a nonlinear oscillator with shape memory alloy using an optimal linear control: Part II: Nonideal energy source. Nonlinear Dyn..

[17] Zuo, X. B., Li, A. Q., Wei Sun & Sun, X. H. Optimal design of shape memory alloy damper for cable vibration control. *JVC J. Vib. Control* **15**, 897–921 (2009).

[18] Ozbulut OE, Roschke PN, Lin PY, Loh CH (2010). GA-based optimum design of a shape memory alloy device for seismic response mitigation. Smart Mater. Struct..

[19] Das, S. & Mishra, S. K. Optimal performance of buildings isolated by Shape-Memory-Alloy-Rubber- Bearing (SMARB) under random earthquakes. *Int. J. Comput. Methods Eng. Sci. Mech.* **15**, 265–276 (2014).

[20] Hassanzadeh A, Moradi S (2022). Topology optimization and seismic collapse assessment of shape memory alloy (SMA)-braced frames: Effectiveness of Fe-based SMAs. Front. Struct. Civ. Eng..

[21] Mulay N, Shmerling A (2021). Analytical approach for the design and optimal allocation of shape memory alloy dampers in three-dimensional nonlinear structures. Comput. Struct..

[22] Das S, Tesfamariam S (2020). Optimization of SMA based damped outrigger structure under uncertainty. Eng. Struct..

[23] Das S, Tesfamariam S (2022). Multiobjective design optimization of multi-outrigger tall-timber building: Using SMA-based damper and Lagrangian model. J. Build. Eng..

[24] Mirzai, N. M., Mansouri, I., Tezcan, J., Awoyera, P. O. & Wan Hu, J. Estimating optimum parameters of a new SMA damper under different earthquake ground motions. in *Structures* vol. 33 2700–2712 (Elsevier, 2021).

[25] Chang, Z., Xing, G., Han, M. & Liu, B. Multiparameter optimization design of self-centering friction damper using shape memory alloy bars. *J. Earthq. Eng.* 1–19 (2022). 10.1080/13632469.2022.2033354.

[26] Wang C, Foliente GC, Sivaselvan MV, Reinhorn AM (2001). Hysteretic models for deteriorating inelastic structures. J. Eng. Mech..

[27] Wilde K, Gardoni P, Fujino Y (2000). Base isolation system with shape memory alloy device for elevated highway bridges. Eng. Struct..

[28] Graesser EJ, Cozzarelli FA (1991). Shape-memory alloys as new materials for aseismic isolation. J. Eng. Mech..

[29] Witting, P. R. & Cozzarelli, F. A. Experimental determination of shape memory alloy constitutive model parameters. in *Active Materials and Smart Structures* vol. 2427 260–275 (International Society for Optics and Photonics, 1995).

[30] Gerdts, M. *Optimal Control of ODEs and DAEs*. *Optimal Control of ODEs and DAEs* (Walter de Gruyter, 2012). 10.1515/9783110249996.

